# A Dual Luminescent and Chromogenic Pt-NCN Complex for the Detection of GTP and CTP in Aqueous Media

**DOI:** 10.1021/acs.inorgchem.5c04987

**Published:** 2026-01-05

**Authors:** Josue Valdes-García, Mireille Vonlanthen, Fabián Cuétara-Guadarrama, David Garcia-Bassoco, Hector Luis Valdés-Negrín, Arturo López-Pérez, Ernesto Rivera, Simón Hernández-Ortega, Alejandro Dorazco-González

**Affiliations:** † Instituto de Investigaciones en Materiales, 7180 Universidad Nacional Autónoma de México, Circuito Exterior, Ciudad Universitaria, C.P. 04510 Ciudad de México, México; ‡ Instituto de Química, 7180 Universidad Nacional Autónoma de México, Circuito Exterior, Ciudad Universitaria, C.P. 04510 Ciudad de México, México

## Abstract

Selective recognition of nucleotides by synthetic receptors in aqueous media is an important area in supramolecular analytical chemistry due to their key biochemical functions. However, selective receptors for GTP and CTP remain an ongoing challenge. Herein, a water-soluble [Pt­(NCN)­Cl] complex (NCN = 1,3-bis­(benzimidazole)­benzene derivative bearing two tetraethylene glycol chains), (**4**), was synthesized, characterized, and structurally described by single-crystal X-ray diffraction. Complex **4** was studied as receptor for the detection of nucleotides, nucleosides, and oxyanions in water at pH = 7.4, showing the greatest binding affinity for GTP (log *K*_1:1_ = 6.85 ± 0.01) via a luminescence turn-off response, with a limit of detection (LOD) = 1.28 μM. Such GTP selectivity/affinity was ascribed to the formation of coordination bond Pt–N7 with a guanosine ring and hydrogen bonds between tetraethylene glycol chains and the triphosphate group. On the other hand, CTP induced a visual color change from colorless to green attributed to the formation of aggregates induced by Pt···Pt and π–π interactions. The logarithmic binding and oligomerization constants for CTP were determined to be 5.32 ± 0.03 and 4.55 ± 0.02, respectively, with a value of LOD = 2.29 μM. The chemosensing mechanisms were investigated by NMR spectroscopy, lifetime resolved fluorescence, Stern–Volmer quenching studies, electrospray mass spectrometry and DFT calculations.

## Graphical abstract

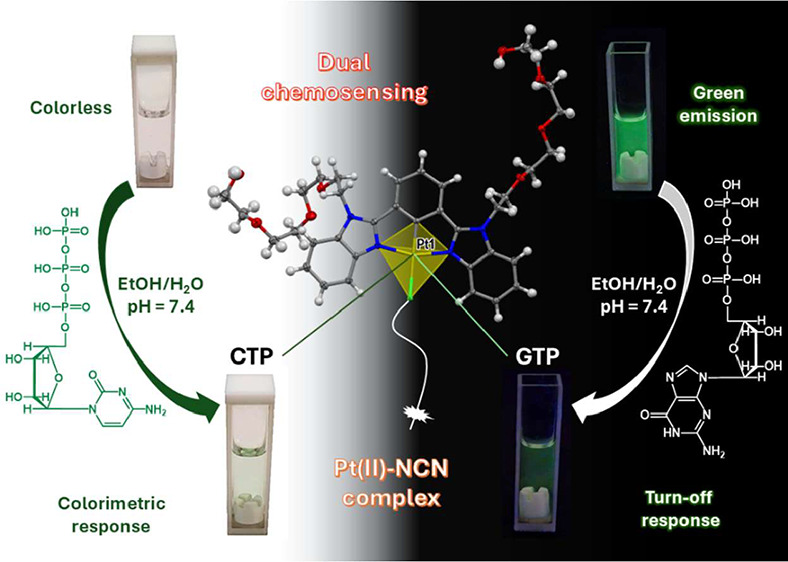

## Introduction

Nucleoside triphosphates (NTPs) play a key role in several biochemical processes.^1^ For instance, guanosine-5′-triphosphate (GTP) is involved in the citric acid cycle, RNA synthesis, protein synthesis, and activation of G-protein.^2^ Cytidine-5′-triphosphate (CTP) participates in brain cell repair, glycerophospholipids synthesis and glycosylation of proteins.^3^ Imbalances in GTP and CTP levels are associated with human diseases, such as cancer, Alzheimer’s disease, heart failure, tuberculosis, and African sleeping sickness, among others.^3–6^ Therefore, in order to gain a deeper comprehension of these essential processes, selective chemosensors need to be developed. Fluorescent and colorimetric chemosensors have been reported for the recognition of NTPs owing to their high sensitivity and rapid response.^7–10^ Since NTPs such as GTP and CTP are present in plasma and other extracellular fluids in concentrations of the micromolar range,^11^ it is necessary to develop selective NTP receptors with the ability to operate in aqueous media at submicromolar concentrations.

Previous studies have demonstrated that selective binding of NTPs is the result of a combination of noncovalent interactions (π–π stacking, hydrogen bonding, and van der Waals forces),^12–15^ coordination bonds,^16,17^ and dynamic covalent bonds (phenylboronic acids).^18^ On the one hand, selective recognition of GTP has been reported using binuclear Zn­(II) complexes,^19–21^ cyclophanes,^22,23^ bisquinolinium compounds,^24^ squaraine dyes,^25^ cationic tentacle porphyrin,^26^ and a tris-naphthoimidazolium derivative.^27^ On the other hand, tris­(hydroxymethyl)­aminomethane derivatives, and benzo­[*de*]­isoquinoline-1,3-diones macrocycles have been studied for selective recognition of CTP.^28,29^ The optical properties of the receptors undergo significant changes upon binding to the NTP nitrogen base and triphosphate motif, typically with binding constants between 10^3^ and 10^6^ M^–1^. However, most of these receptors are blue-emitting fluorescent chemosensors whose optical responses can be significantly affected by light scattering and background fluorescence. To minimize these interferences, phosphorescent chemosensors, such as lanthanide complexes, have been developed due to their long luminescence lifetimes (≥10^–6^ s).^30^

Thanks to the strong oxophilic character of lanthanide metals, these receptors can bind the NTP triphosphate motif through the metal center. However, one major drawback of lanthanide-based receptors is their low selectivity and affinity, as the metal center is the only binding site.^31^ Thus, lanthanide-based receptors, incorporating a platinum metal center as a second and cooperative binding site, have demonstrated enhanced affinity for CTP and GTP by forming complexes between the Pt­(II) atom and N-donor ligands of the nitrogen base.^32^

In this regard, a heterotrinuclear Tb­(III)–Pt­(II) complex has demonstrated superior selectivity toward guanine nucleotides, which was attributed to the coordination of the Pt­(II) atom with N7 atom from guanine ring. The sensing mechanism comprised luminescence enhancement of the receptor through energy transfer, from guanine to Tb­(III), with partial exclusion of coordinated water molecules.^33^ Another example involved DNA recognition with heterotrinuclear Eu­(III)–Pt­(II) and heterodinuclear Ru­(II)–Pt­(II) complexes by intercalation with the DNA nucleobases.^34,35^

Organometallic Pt­(II)-NCN complexes exhibit a structured emission band at ca. 500–550 nm ascribed to mixed triplet excited states of intraligand (^3^IL) and metal-to-ligand charge-transfer (^3^MLCT) with lifetimes in the microsecond range.^36,37^ These complexes are prone to associate with each other in the ground/excited state by Pt···Pt and/or π–π interactions.^38^ Moreover, the emission wavelength, lifetime, and quantum efficiency of Pt­(II)-NCN complexes can be modified by changing the coordination environment of the Pt­(II) or the structure of the ligand, making them attractive in chemosensing applications.^39–43^ In this line, Gong and Zhong reported a [Pt­(NCN)­Cl] complex functionalized with an amide group able to selectively interact with H_2_PO_4_^–^ through hydrogen-bonding inducing color changes from green to yellow in DCM and green to red in MeCN.^44^ This receptor is only soluble in organic solvents, which seriously limits its applications in biological systems. To improve the solubility in aqueous media, a variety of hydrophilic functional groups have been used to obtain amphiphilic organometallic compounds. For example, Yam and co-workers have reported a class of amphiphilic anionic Pt­(II)-NNN complexes bearing propanesulfonate motifs. The complexes featured high solubility, stability, strong emission in water, and controlled supramolecular self-assembly via Pt···Pt aggregation and π–π interactions by varying solvent composition.^45^ Similarly, oligo­(ethylene glycol) motifs have been incorporated in Au­(III)-NCN complexes to confer stability, solubility, and supramolecular self-assembly in aqueous solution.^46^ Hou and co-workers reported an amphiphilic gemini-Ir­(III) complex bearing oligo­(ethylene glycol) groups for rapid and selective detection of picric acid through luminescence quenching by photoinduced electron transfer (PET) and resonance energy transfer mechanisms.^47^

However, chemical sensing of NTPs with Pt­(II)-NCN complexes in aqueous media remains largely unexplored. In this work, an amphiphilic organometallic Pt­(II)-NCN complex bearing tetraethylene glycol moieties (**4**) as a selective chemosensor for GTP and CTP is presented. The synthesis, characterization, and crystal structure of the Pt-NCN complex is reported as well as its optical properties in aqueous solution. Sensing studies by spectroscopy revealed that receptor **4** was capable of sensing GTP via a luminescence turn-off response, whereas CTP led to a color change from colorless to green together with the formation of aggregates. The binding constants for GTP and CTP were determined. Additionally, the oligomerization constant for CTP was estimated. Furthermore, the limit of detection (LOD) for all cases was calculated. The coordination behavior of **4** with GTP and CTP was supported by ^1^H and ^31^P NMR, fluorescence lifetimes, Stern–Volmer quenching studies, electrospray mass spectrometry, and DFT calculations.

## Experimental Section

### General Conditions

All reagents, solvents, and techniques are described in the Supporting Information. Compounds 1,3-bis­(1H-benzo­[*d*]­imidazol-2-yl)­benzene (**1**) and 2-(2-(2-(2-bromoethoxy)­ethoxy)­ethoxy)­ethan-1-ol (**TEG-Br**) were synthesized following previously reported methods.^48,49^

### Synthesis of NCN Ligand, **2**

Compound **1** (56.1 mg, 0.18 mmol) was dissolved in dry DMF (5 mL) and stirred with Cs_2_CO_3_ (123.8 mg, 0.38 mmol) at room temperature (rt) for 1 h. A solution of **TEG-Br** (123.8 mg, 0.43 mmol) in dry DMF (1 mL) was added, and the reaction mixture was heated to 80 °C and left stirring overnight under nitrogen atmosphere. After cooling, the reaction mixture was filtered through Celite, and the solvent was removed under reduced pressure. The crude product was purified by column chromatography (AcOEt to AcOEt/MeOH (21:4 v/v)), obtaining compound **2** as a yellow oil. Yield: 75% (89.5 mg). ^1^H NMR (300 MHz, 298 K, CDCl_3_, δ): 8.28 (s, 1H), 8.01 (dd, *J* = 7.8, 1.7 Hz, 2H), 7.85–7.80 (m, 2H), 7.70 (t, *J* = 7.8 Hz, 1H), 7.53–7.47 (m, 2H), 7.35–7.29 (m, 4H), 4.51 (t, *J* = 5.4 Hz, 4H), 3.85 (t, *J* = 5.4 Hz, 4H), 3.63 (t, *J* = 5.5 Hz, 4H), 3.49–3.41 (m, 16H), 3.34–3.01 (m, 4H), 3.01 (br s, 2H, OH). ^13^C NMR (75.5 MHz, 298 K, CDCl_3_, δ): 153.5, 142.5, 135.6, 131.5, 131.1, 130.7, 129.2, 123.2, 122.9, 119.8, 110.6, 72.6, 70.8, 70.5, 70.3, 70.2, 69.1, 61.5, 45.0. ESI­(+)-MS (*m*/*z*): calculated for C_36_H_47_N_4_O_8_ [**2** + H]^+^, 663.79; found 663.34. ATR-IR (cm^–1^): ν = 3329.10 (w), 2866.80 (m), 1456.26 (m), 1098.48 (s), 1066.13 (s), 746.51 (s). Elemental analysis calcd for C_36_H_46_N_4_O_8_ (%): C, 65.24; H, 7.00; N, 8.45; O, 19.31. Found: C, 64.38; H, 6.97; N, 8.34.

### Synthesis of Pt­(II)-NCN Complex, **3**

A solution of compound **2** (81.4 mg, 0.12 mmol) and K_2_[PtCl_4_] (61.7 mg, 0.14 mmol) in 30 mL of acetic acid was bubbled with N_2_ and heated to 120 °C for 24 h under a nitrogen atmosphere. After this time, the solvent was removed under reduced pressure. The crude product was taken in chloroform, filtered through Celite, and the solvent was removed under reduced pressure. The crude was purified by column chromatography (AcOEt to AcOEt/MeOH (23:2 v/v)) to obtain product **3** as a brown oil. Yield: 72% (86.0 mg). ^1^H NMR (300 MHz, 298 K, CDCl_3_, δ): 8.85 (d, *J* = 7.9 Hz, 2H), 7.37 (d, *J* = 7.8 Hz, 2H), 7.29–7.26 (m, overlapped with residual CHCl_3_, 2H), 7.23–7.11 (m, 4H), 7.06 (t, *J* = 7.8 Hz, 1H), 4.64 (t, *J* = 5.4 Hz, 4H), 4.15–4.12 (m, 4H), 3.87 (t, *J* = 5.4 Hz, 4H), 3.60–3.56 (m, 4H), 3.47–3.41 (m, 8H), 3.39–3.34 (m, 8H), 2.02 (s, 6H). ^13^C NMR (75.5 MHz, 298 K, CDCl_3_, δ): 171.1, 161.5, 161.2, 141.2, 134.4, 131.6, 124.3, 123.0, 122.5, 119.1, 109.7, 71.1, 70.6, 70.5, 69.4, 69.1, 63.6, 45.0, 21.0. ESI­(+)-MS (*m*/*z*): calculated for C_40_H_49_N_4_O_10_Pt [**3** – Cl^–^]^+^, 940.93; found 940.31. ATR-IR (cm^–1^): ν = 2900.07 (w), 2866.32 (w), 1730.63 (s), 1440.79 (m), 1242.04 (m), 1122.36 (s), 1052.97 (s), 740.95 (s), 439.54 (m). Elemental analysis calcd for C_40_H_49_ClN_4_O_10_Pt (%): C, 49.21; H, 5.06; Cl, 3.63; N, 5.74; O, 16.39; Pt, 19.98. Found: C, 49.74; H, 5.92; N, 5.86.

### Synthesis of Amphiphilic Pt­(II)-NCN Complex, **3**

To a solution of **3** (80 mg, 0. 08 mmol) in 10 mL of MeOH:DCM (1:9, v/v), KOH (9. Two mg, 0.16 mmol) was added, and the reaction mixture was stirred overnight at r.t. Afterward, the solvent was removed under reduced pressure. The crude product was dissolved in CHCl_3_ and filtered. Afterward, the solvent was removed under reduced pressure to give compound **4** as a yellow solid. Yield: 78% (57.2 mg). ^1^H NMR (300 MHz, 298 K, CDCl_3_, δ): 8.83–8.80 (m, 2H), 7.38 (dd, *J* = 7.7, 3.8 Hz, 2H), 7.28 (m, overlapped with residual CHCl_3_, 2H), 7.20 (m, 4H), 7.03 (t, *J* = 7.7 Hz, 1H), 4.64 (t, *J* = 5.5 Hz, 4H), 3.85 (t, *J* = 5.4 Hz, 4H), 3.61 (m, 4H), 3.49–3.28 (m, 22H). ^13^C NMR (75.5 MHz, 298 K, CDCl_3_, δ): 161.5, 161.0, 141.2, 134.4, 131.5, 124.4, 124.3, 123.0, 122.5, 119.0, 109.8, 72.4, 71.1, 70.6, 70.4, 70.1, 69.3, 61.6, 44.9. ESI­(+)-MS (*m*/*z*): calculated for C_38_H_49_N_5_O_8_Pt [**4** – Cl^–^ + MeCN]^+^, 898.91; found 898.30. ATR-IR (cm^–1^): ν = 3391.40 (w), 2863.77 (m), 1441.38 (m), 1096.75 (s), 745.84 (s), 721.74 (s), 437.67 (w). Elemental analysis calcd for C_36_H_45_ClN_4_O_8_Pt (%): C, 48.46; H, 5.08; Cl, 3.97; N, 6.28; O, 14.34; Pt, 21.86. Found: C, 47.83; H, 4.90; N, 5.77.

### Fluorometric and Spectrophotometric Studies

Stock solutions (0.125 mM) of nucleotides, nucleosides, and oxyanions were prepared in Milli-Q water for fluorometric studies. Fluorometric titration experiments were performed by adding aliquots to aqueous MOPS/EtOH buffer solutions (7:3, 10 mM, pH = 7.4) containing compound **4** (3 μM), reaching a final analyte concentration of 6 μM. For UV–vis titration experiments, aliquots of 0.5 mM stock solutions were added to the same buffer solutions containing compound **4** (15 μM), to reach a final analyte concentration of 45 μM.

### ^1^H and ^31^P NMR Studies

^1^H and ^31^P NMR (300 and 121.5 MHz, respectively) experiments were recorded to solutions for GTP (3.82 mM) and CTP (3.80 mM) in 0.5 mL of D_2_O, before and after the addition of 1 and 2 equiv of compound **4.**

### Crystallographic Experimental

Suitable crystals were coated with hydrocarbon oil, picked up with a nylon loop, and mounted under the cold nitrogen stream in the diffractometer, using MoKa (*k* = 0.71073 Å) radiation on a Bruker APEXIII CCD with a microfocus X-ray source and Helios multilayer mirror.^50^ Semiempirical absorption correction was applied to all data sets. Structure solutions, refinements, and geometrical calculations have been carried out by SHELXTL.^51^ The structure was solved by direct methods and refined using the full-matrix least-squares on F2 with all non-H atoms anisotropically defined.^51,52^ The hydrogen atoms were placed in calculated positions using the “riding model” with *U*_iso_ = *a* × *U*_eq_ (where *a* is 1.5 for −CH3 and −OH moieties and 1.2 for others). Crystallographic data for compound **4** have been deposited at the Cambridge Crystallographic Data Center under CCDC 2465219 and can be freely obtained from https://www.ccdc.cam.ac.uk/structures.

### DFT Calculations

All quantum chemical calculations were performed with Gaussian 16 (Revision A.03).^53^ Geometry optimization was performed using the hybrid density functional B3LYP with Grimme’s DFT empirical dispersion correction with Becke–Johnson damping (DFT-D3­(BJ)).^54^ This model accounts for long-range dispersion interactions that are essential to describe noncovalent interactions. The split-valence polarized basis set 6-31G­(d,p) was employed for the main-group elements (H, C, O, N, and P); while LANL2DZ and MWB6 effective core potentials were used for Na and Pt atoms, respectively. All optimized geometries were confirmed to be a true minimum on the potential energy surface through harmonic vibrational frequency calculations by the absence of imaginary frequencies. Complexation energies were obtained with the Boys–Bernardi correction for basis set superposition error (BSSE) as implemented in Gaussian 16 (keyword counterpoise).^55,56^ The corrected complexation energies were calculated according to eq 1

$$\Delta E_{\mathrm{int},\;\mathrm{AB}}^{\mathrm{CP}}=E_{\mathrm{AB}}^{\mathrm{AB}}(\mathrm{AB})-[E_{A}^{\mathrm{AB}}(\mathrm{AB})+E_{B}^{\mathrm{AB}}(\mathrm{AB})]$$ 1

where *E*_AB_^AB^(AB) is the interaction energy of the AB complex, evaluated in the full AB basis set; *E*_A_^AB^(AB) is the energy of fragment A in the complex, evaluated in the full AB basis (ghost atoms on fragment B); *E*_B_^AB^(AB) is the energy of the fragment B in the complex, evaluated in the full AB basis (ghost atoms on fragment A). The wave functions of the optimized complexes were further analyzed using Quantum Theory of Atoms in Molecules (QTAIM) and noncovalent interaction (NCI) index implemented in Multiwfn software version 3.8.^57–61^ The resulting molecular graphs and NCI isosurfaces were rendered using VMD visualization software version 1.9.3 with the Tachyon parallel ray tracing library.^62^

## Results and Discussion

### Synthesis, Crystal Structure, and Hirshfeld Surface Analysis

The amphiphilic Pt­(II)-NCN complex (**4**) was successfully synthesized as depicted in Scheme 1. Compound **1** was reacted with Cs_2_CO_3_ in dry DMF, followed by the addition of **TEG-Br** to give compound **2**. Direct metalation of **2** with K_2_[PtCl_4_] in AcOH under a N_2_ atmosphere yielded compound **3**. Finally, receptor **4** was obtained by hydrolysis of compound **3** in a basic medium (KOH in MeOH:DCM). The characterization of all the synthesized compounds including ^1^H and ^13^C NMR and ATR-IR spectroscopies and positive scan MS-ESI are included in the Supporting Information (Figures S1–S14). Complex **4** was obtained in high purity as evidence by ^1^H and ^13^C NMR, MS-ESI (positive scan) and ATR-IR spectroscopies (Figures S11–S14). A charged state of Pt­(II)-NCN complex **4** was observed at 898.30 *m*/*z* by the MS-ESI positive scan, which corresponded to the cationic species [**4** – Cl^–^ + MeCN]^+^. The peak multiplicity matches the theoretical distribution for complex **4** (without chloride and a molecule of MeCN), see Figure S13.

**Scheme 1:**
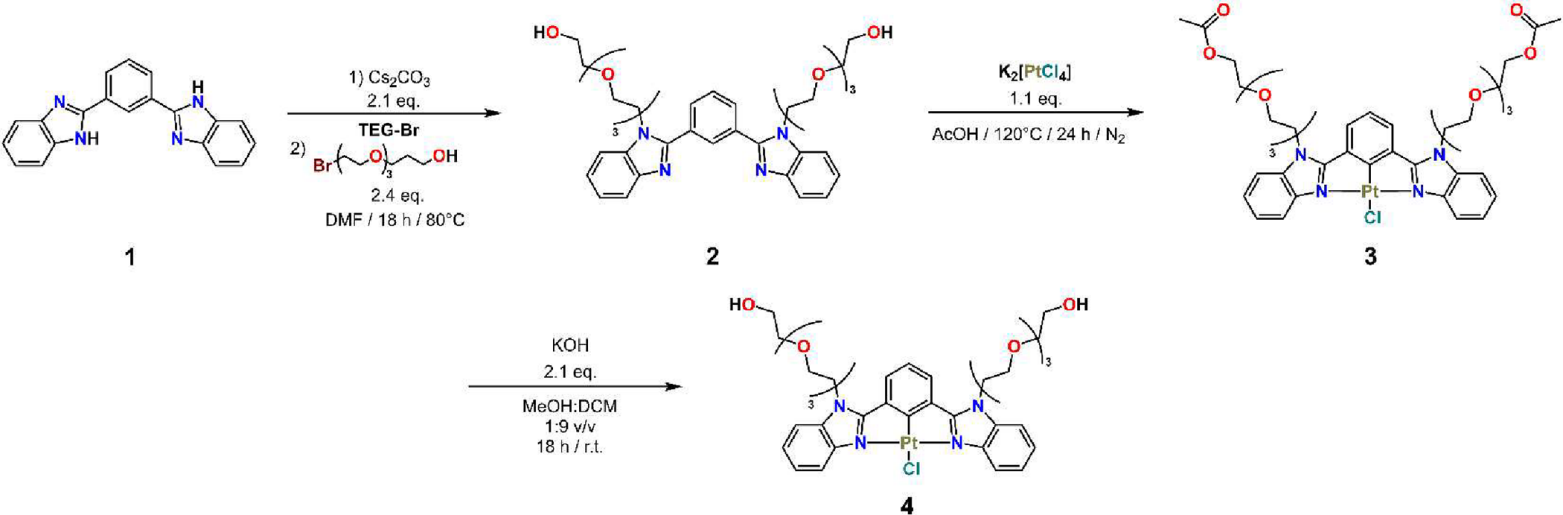
Synthesis of Amphiphilic Pt­(II)-NCN Complex, **4**

A crystalline sample of complex **4** was obtained by cold crystallization from EtOH (Figure 1A). The X-ray crystallographic data of complex **4** including selected distances/angles around the Pt­(II) atom and hydrogen bond parameters within the crystal packing are included in Tables S1–S3 of the Supporting Information.

**Figure 1:**
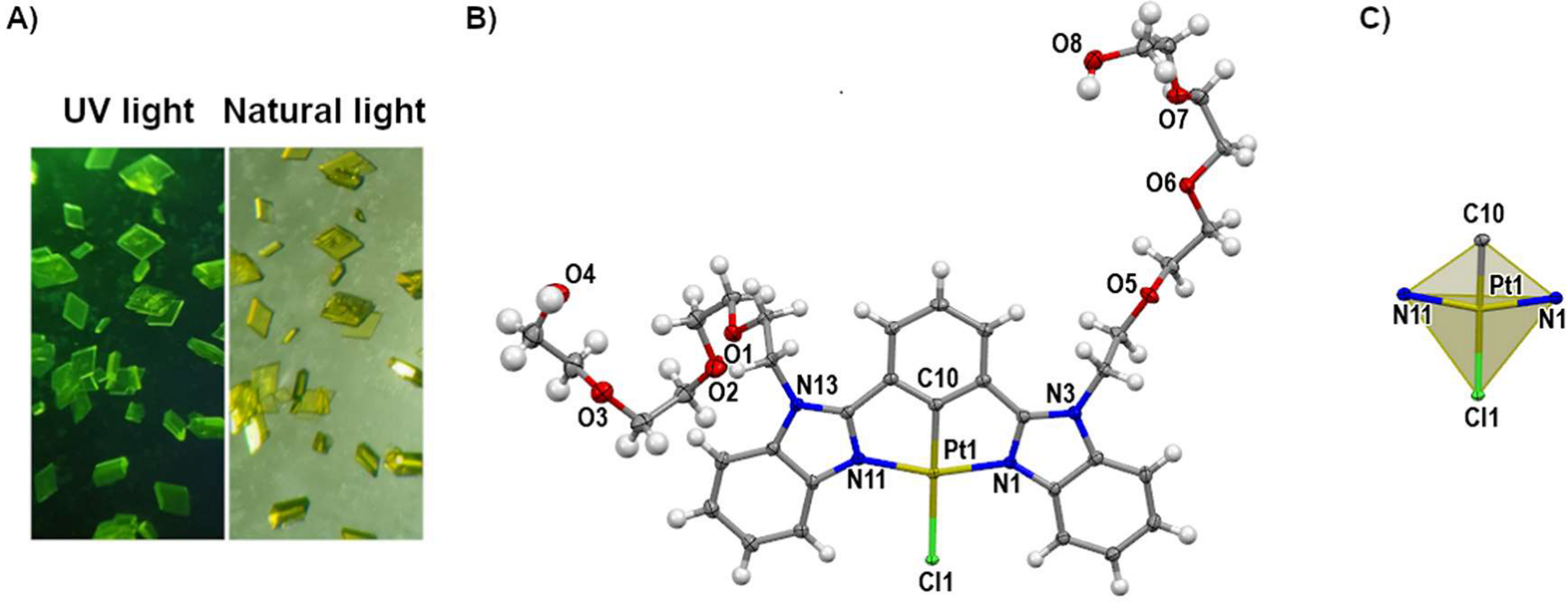
(A) Image of a crystal of 4 under UV light (left) and natural light (right) irradiation. (B) Perspective view of the X-ray molecular structure of amphiphilic complex, **4** (numbering of important atoms is depicted). (C) Representation of the geometry around the Pt­(II) atom (the ellipsoids are 50% probability).

A perspective of the molecular structure of **4** obtained from X-ray diffraction data is shown in Figure 1B. Crystallographic analysis of complex **4** showed a Pt­(II) metal center adopting a distorted-square-planar geometry (Figure 1C and Table S2). The Pt–Cl bond distance (2.4262(5)­Å) is significantly longer than that in previously reported Pt-NNN complexes such as [Pt­(N­(H)­dpa)­Cl]­H_2_PO_4_ (2.3148(6) Å),^63^ [Pt­(terpy-COOH)­Cl]­BF_4_ (2.302(2)­Å),^64^ [Pt­(4′-(2-quin)-terpy)­Cl]­(PF_6_)_2_ (2.3046(10)­Å).^65^ The longer Pt–Cl bond distance found for complex **4** can be attributed to the strong *trans* influence of C atom (C10) opposite to the chloride atom.^39,43,66,67^

Crystal packing of amphiphilic complex **4** is shown in Figure 2. An antiparallel head-to-tail dimer formed via π–π stacking interactions (3.87 Å) is shown in Figure 2A. Despite tetraethylene glycol (TEG) motifs adopting a *syn* conformation, the displacement of the antiparallel dimer yields an intermolecular Pt···Pt distance of 4.96 Å, precluding Pt···Pt interactions. One TEG chain extends along the *b*-axis through π–π stacking interactions with adjacent benzimidazole units and a centroid-centroid of 3.67 Å (Figure S15A).

**Figure 2:**
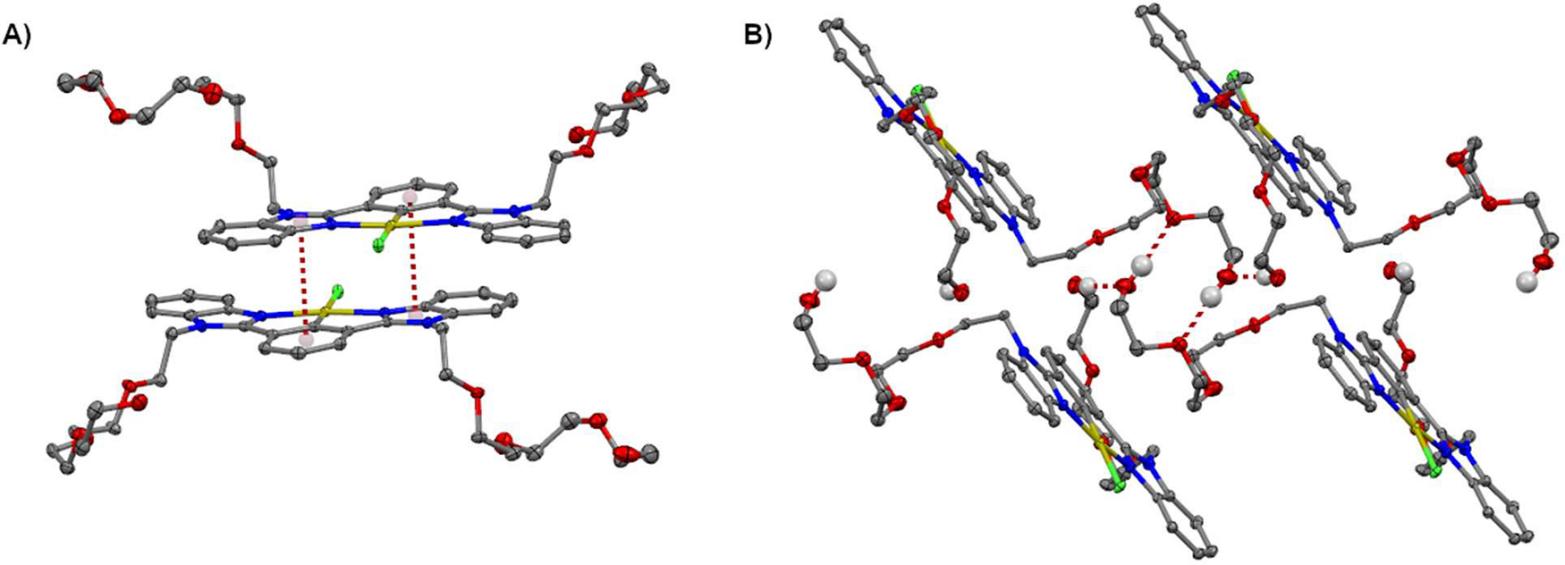
(A) Dimer of 4 formed by π–π stacking interaction (distance = 3.87 Å). (B) Dimeric *R*_2_^2^(10) homosynthon observed in **4**. Only the relevant hydrogen atoms are shown for the sake of clarity.

The crystal structure of **4** displays intermolecular OH···O hydrogen bonds (H-bonds) as shown in Figure 2B. The hydrogen bonds are formed via a dimeric *R*_2_^2^(10) homosynthon in which hydroxyl groups adopt *syn*-*syn* conformation. An additional H-bond is formed between homosynthon and the hydroxyl group of one of the TEG motifs. A 1D chain along the *c*-axis is stabilized by H-bonds involving dimeric *R*_2_^2^(10) homosynthons (Figure S15B). Similarly, a 1D chain along the *a*-axis was formed through weak interaction of the type C–H···Cl with a 2.93 Å distance (Figure S15C).

Hirshfeld surface analysis and 2D-fingerprint plots revealed H-bonds and π–π stacking interactions. In the surface mapped over *d*_norm_, the important H-bonds are depicted as circular red spots, as is illustrated in Figure 3A. Intense red regions indicate intermolecular H-bonds between TEG motifs of the type O–H···O. Additionally, π–π stacking interactions are depicted as red/blue triangles on the shape-index surface, as shown in Figure 3B. The 2D-fingerprint plots (Figure S16) were obtained for the quantitative analysis of the intermolecular interactions within the crystal. The fingerprint plots show that the greatest contribution come from H···H interactions (57.8%), followed by O···H/H···O (14.7%), C···H/H···C (11.4%), Cl···H/H···Cl (5.3%) and C···C/C···C interactions (3.7%). Therefore, crystal packing of complex **4** is mainly stabilized by H···H, O···H/H···O, and C···H/H···C contacts.

**Figure 3:**
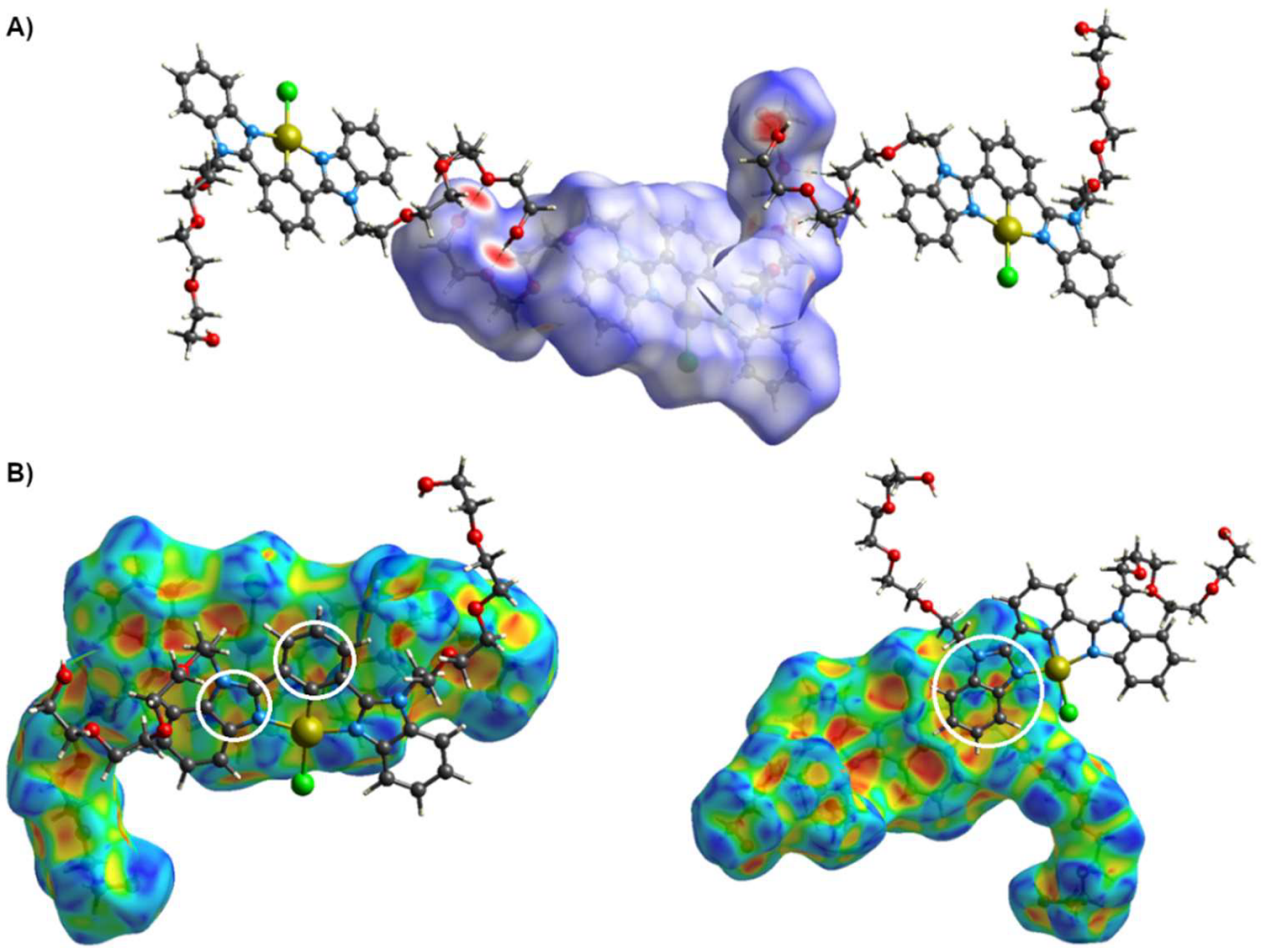
(A) Hirshfeld surface mapped over *d*_norm_ of the complex **4** illustrating H-bonds. (B) Hirshfeld surface mapped with the shape index of amphiphilic complex **4**, and π–π stacking interactions are highlighted inside white circles.

### Optical Properties

Complex **4** was studied by UV–vis and fluorescence spectroscopies in aqueous MOPS/EtOH buffer solution (7:3, 10 mM, pH = 7.4). The absorption linearity was found up to a concentration of 30 μM, whereas the emission linearity was found below 10 μM, possibly owing to self-quenching of luminescence in Pt­(II) complexes.^67–69^ Therefore, the concentration of complex **4** within these ranges was suitable for further photophysical studies. Moreover, the addition of EtOH was necessary to obtain reproducible titration results by increasing the solubility of complex **4** in the presence of the NTPs.

Absorption and emission maxima (λ_exc_ = 380 nm) values of **4** in aqueous MOPS/EtOH buffer solution (7:3, 10 mM, pH = 7.4) are presented in Table S4 and the corresponding spectra are shown in Figure S17. Complex **4** displayed an intense absorption band between 266–336 nm, which was assigned to the NCN IL [π → π*] transition (compound **2**), and low-energy absorption band between 336–450 nm which was ascribed to mixed transitions of IL [π → π*] and [dπ­(Pt) → π*­(**2**)] transitions.^37,70^ Upon excitation at 380 nm, a structured emission band was observed in the range between 450–670 nm. According to previously reported Pt­(II)-NCN complexes, the emission of complex **4** originates from the ^3^IL [π → π*]/^3^MLCT [dπ­(Pt) → π*­(**2**)] excited state.^39,66,71^ The quantum yield of **4** was calculated in an air-equilibrated solution using quinine sulfate (Φ_f_ = 0.54 in 0.1 M H_2_SO_4_)^72^ according to eq 2:

$$\Phi_{x}=\Phi_{\mathrm{st}}\left(\frac{{\mathrm{grad}}_{x}}{{\mathrm{grad}}_{\mathrm{st}}}\right)\left(\frac{\eta_{x}^{2}}{\eta_{\mathrm{st}}^{2}}\right)$$ 2

where the subscripts st and x denote standard and sample, respectively. Φ is the quantum yield, grad is the gradient from the plot of integrated fluorescence intensity vs absorbance, and η is the refractive index of the solvent. The obtained value Φ_f_ = 0.018 was similar to that of a previously reported Pt­(II)-NCN complex, Φ_f_ = 0.01.^73^

### Fluorometric GTP Recognition

To evaluate the ability of Pt­(II)-NCN complex **4** as a luminescent chemosensor for NTPs, we carried out a selectivity experiment by fluorescence spectroscopy. For that purpose, different analytes such as NTPs, nucleosides, sodium pyrophosphate (PPi), NaAcO, and NaH_2_PO_4_ ([analyte] = 3 μM) were added to an aqueous MOPS/EtOH buffer solution (7:3, 10 mM, pH = 7.4) of receptor **4** (3 μM). The percentage of emission change calculated at λ_em_ = 500 nm and the emission spectra of receptor **4** after the addition of analytes are shown in Figure 4A,B, respectively. A photograph of the observed fluorescence of receptor **4** in the presence of the studied analytes is shown in Figure 4C. Chemical structures of the analytes studied are shown in Figure 4D.

**Figure 4:**
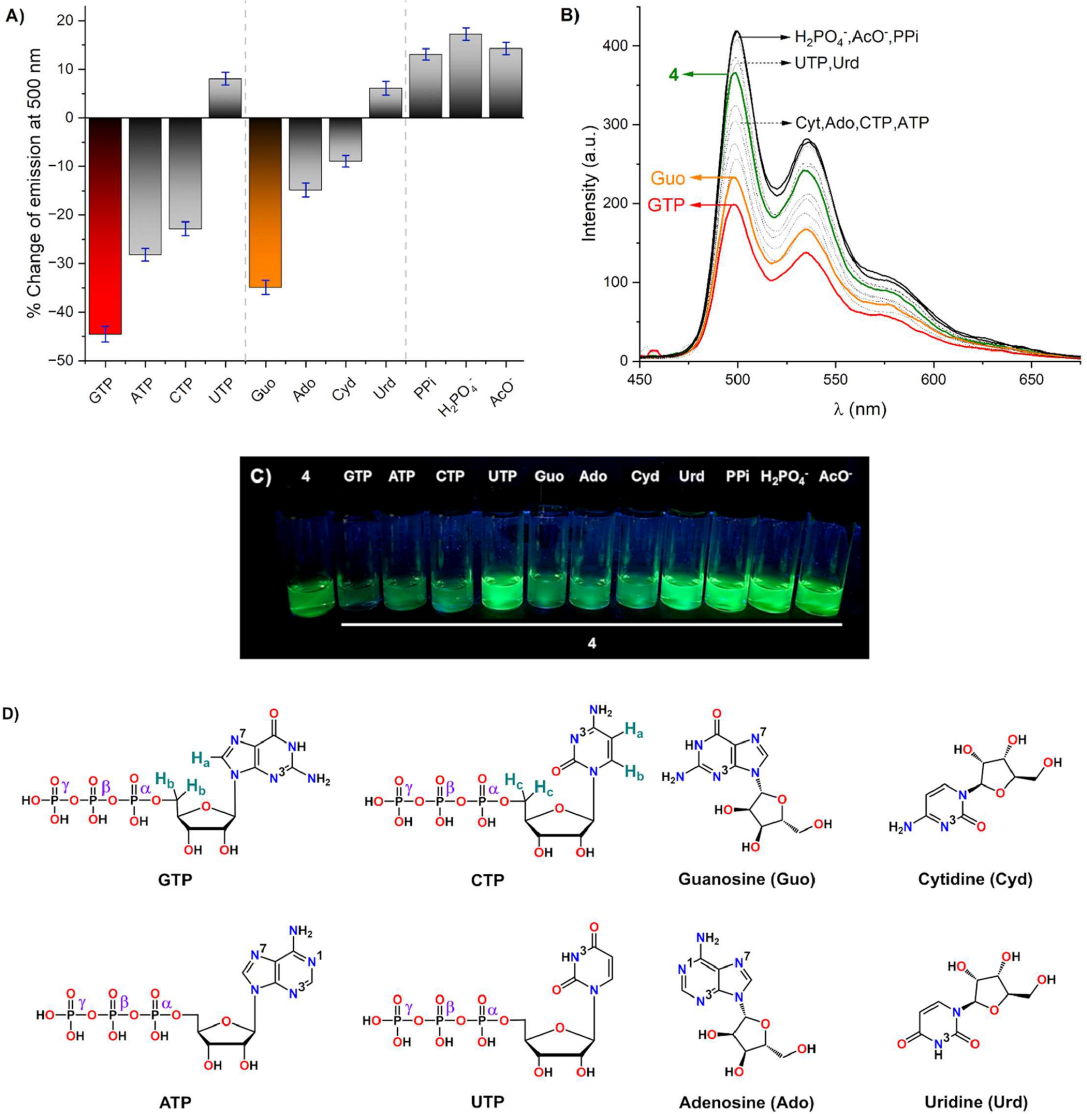
(A) Emission change percentage of receptor **4** calculated at 500 nm upon addition of NTPs, nucleosides, and oxyanions. (B) Emission spectra of receptor **4** after the addition of analytes in aqueous MOPS/EtOH buffer solution (7:3, 10 mM, pH = 7.4). (C) Photograph of solutions of receptor **4** with different analytes under UV light. (D) Chemical structures of NTPs and nucleosides used in this study.

Addition of GTP, ATP, and CTP to solutions of receptor **4** reduced the emission intensity by 44, 28, and 23%, respectively. Interestingly, their respective nucleosides presented less marked optical responses with emission intensity reduction values of 35, 15, and 9%, for Guo, Ado, and Cyd, respectively. These results strongly suggest that the optical change of **4** was produced by the nitrogenous base present, becoming the main recognition site. Additionally, the difference in emission intensity values of GTP, ATP, and CTP with respect to their corresponding nucleosides can be rationalized by the presence of a specific polyphosphate recognition motif in receptor **4**. Conversely, UTP and Urd slightly increased the emission intensity by 8 and 6%, respectively. Possibly, UTP and Urd bind with receptor **4** by H-bonding since the Pt­(II) atom cannot interact with the N3-atom of the nitrogenous base due to the presence of the H3 atom (see Figure 4D for atom labeling). Among the NTPs studied, GTP induced the greatest emission change via a turn-off response. Likewise, oxyanions exhibited an emission increment of 13–17%, associated with H-bond interactions. These outputs were consistent with similar receptors used for chemosensing of AcO^–^,^74,75^ PPi,^76^ and H_2_PO_4_^–^.^44,77^

The binding affinities were then determined by fluorometric titration experiments to gain further insight into the affinity of receptor **4** for NTPs and nucleosides. To obtain the *apparent* binding constant (*K*_1:1_), fluorometric titration curves (Figures 5A and 6C) were fitted to a 1:1 binding model by nonlinear least-squares treatment using eq 3:

$$I_{\mathrm{obs}}=I_{R}+0.5\Delta I_{\infty }\left\{[A{]}_{T}+[R{]}_{T}+\frac{1}{K}-{\left[{\left([A{]}_{T}+[R{]}_{T}+\frac{1}{K}\right)}^{2}-[4][R{]}_{T}[A{]}_{T}\right]}^{0.5}\right\}$$ 3

where *I*_obs_ is the observed intensity, *I*_R_ is the intensity of the receptor, Δ*I*_∞_ is the maximum intensity change induced by the presence of the analyte at saturation, [*A*]_T_ and [*R*]_T_ are the total concentration of the analyte and chemosensor, respectively, and *K* is the *apparent* binding constant. Moreover, the 1:1 binding model was confirmed by a Job′s plot between receptor **4** and GTP (Figure S18). Furthermore, Stern–Volmer quenching constants (*K*_SV_) were calculated by fitting *I*_o_/*I* vs [Analyte] line plots (Figure 5D) with eq 4:

$$I_{o}/I=1+K_{\mathrm{SV}}[Q]$$ 4

where *I*_o_ is the emission intensity in the absence of quencher, *I* is the emission intensity in the presence of quencher, *K*_SV_ is the Stern–Volmer constant, and [*Q*] is the concentration of quencher.

**Figure 5:**
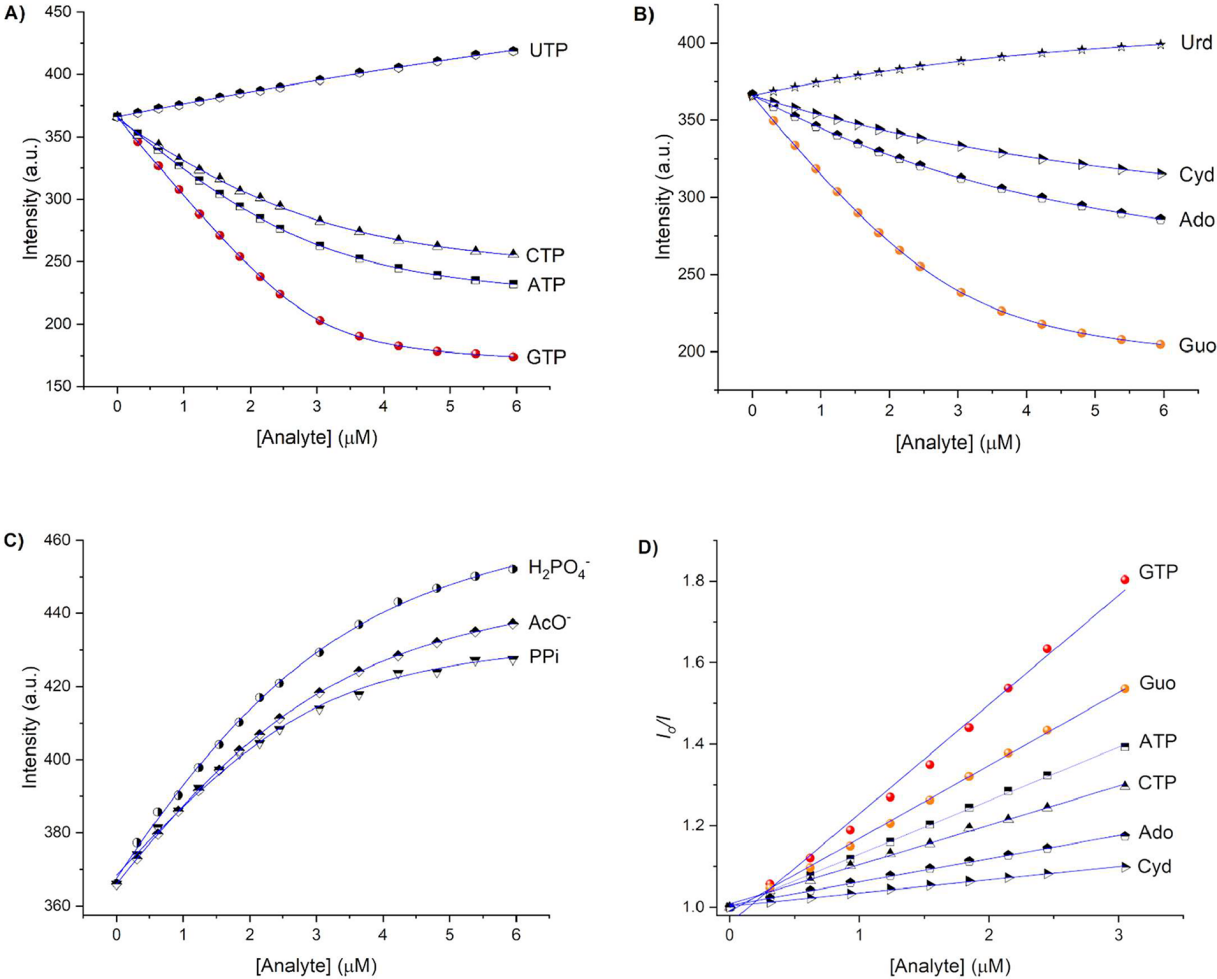
Fluorometric titration (λ_exc_ = 380 nm) of receptor **4** (3 μM) at λ_em_ = 500 nm upon the addition of (A) NTPs, (B) nucleosides and (C) oxyanions in aqueous MOPS/EtOH buffer solution (7:3, 10 mM, pH = 7.4). The curves were fitted using eq 2. (D) Stern–Volmer profiles obtained from fluorometric titrations with NTPs and their nucleosides. The curves were fitted using eq 3.

**Figure 6:**
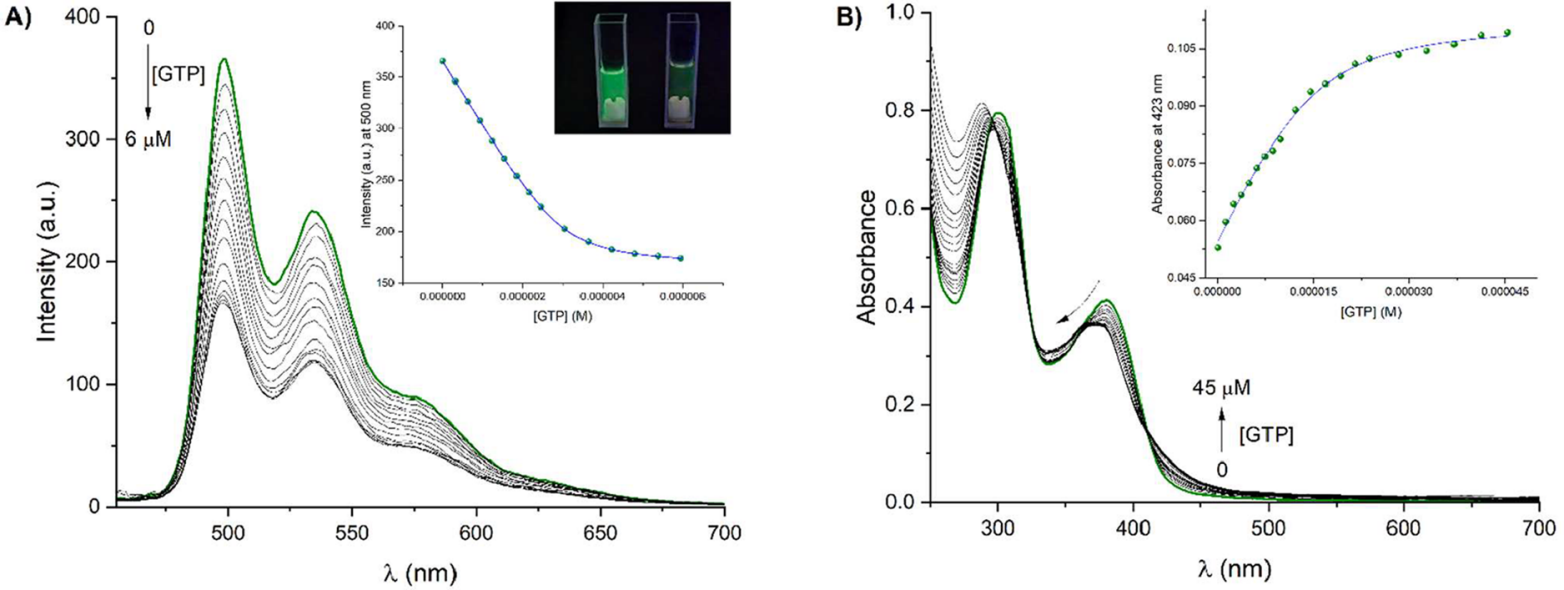
(A) Changes in the emission spectra (λ_exc_ = 380 nm) of receptor **4** (3 μM) upon the addition of GTP (0–6 μM). (B) UV–vis titration of receptor **4** (15 μM) upon the addition of GTP (0–45 μM). Both studies were in aqueous MOPS/EtOH buffer solution (7:3, 10 mM, pH = 7.4). The curves were fitted using eq 2.

The *K*_1:1_ and *K*_SV_ values determined by fluorescence titration are listed in Table 1. As shown in Table 1, GTP exhibited the largest *K*_1:1_ and *K*_SV_ values among all of the studied analytes. Based on the estimated binding constants, the order of binding affinity values of **4** with the studied analytes was GTP> Guo> PPi> ATP> CTP> AcO^–^ ≈ H_2_PO_4_^–^.

**Table 1:** Logarithm of *Apparent* Binding Constants *K*_1:1_ and Quenching Constants *K*_SV_ for Receptor **4** (3 μM) with NTPs as Disodium Salt, Nucleosides, and Oxyanions in Aqueous MOPS/EtOH Buffer Solution (7:3, 10 mM, pH = 7.4)^a^

analyte	log *K*_1:1_	log *K*_SV_	analyte	log *K*_1:1_	log *K*_SV_	analyte	log *K*_1:1_
GTP	6.85 ± 0.01	5.35 ± 0.01	guanosine	6.41 ± 0.01	5.23 ± 0.01	AcO^–^	6.03 ± 0.02
ATP	6.27 ± 0.01	5.12 ± 0.01	adenosine	5.58 ± 0.02	4.76 ± 0.01	PPi	6.32 ± 0.03
CTP	6.11 ± 0.02	4.99 ± 0.01	cytidine	5.57 ± 0.03	4.52 ± 0.01		
UTP	4.56 ± 0.03	NC	uridine	5.39 ± 0.03	NC	H_2_PO_4_^–^	6.00 ± 0.03

a NC = not calculated.

Fluorescence and absorption titration experiments of receptor **4** with GTP are depicted in Figure 6A,B, respectively. The emission intensity diminished by a factor of 2, and a blue shift of the emission band was observed (Δλ = 2 nm) when the concentration of GTP reached 6 μM (Figure 6A). With the fluorescence titration results, we calculated the limit of detection (LOD) via LOD = 3s/S (where s is the standard deviation of the blank and S represents the slope of the calibration curve), obtaining a value of LOD = 1.28 μM.

Spectrophotometric titration experiment of receptor **4** (15 μM) with GTP (Figure 6B) resulted in a gradual decrease of the band intensity at 380 nm and a hypsochromic shift (Δλ = 10 nm). Three isosbestic points at 325, 363, and 411 nm were observed, which indicated that only two species were in chemical equilibrium, receptor **4** and **4-GTP** supramolecular complex. The estimated binding constant by UV–vis at 423 nm (log *K*_1:1_ = 6.23 ± 0.03) was similar to that found by emission, suggesting that complexation of **4** with GTP is mainly formed in the ground state. The resulting solution from UV–vis titration was analyzed by HRMS-ESI­(+) (Figure S19). One species was observed at 1425.3347 *m*/*z* with an isotopic distribution that corresponded to [**4** – Cl^–^ + GTP – 2H^+^ + 2Na^+^]^+^ species, confirming the formation of a 1:1 supramolecular complex.

To analyze the quenching process between receptor **4** and GTP, lifetimes of receptor **4** in the absence and presence of GTP were measured in an aqueous MOPS/EtOH buffer solution (7:3, 10 mM, pH = 7.4) as shown in Figure 7. Receptor **4** displayed a triexponential decay in aqueous media. The τ_1_ and τ_2_ lifetime values were in the order of nanoseconds (0.51 and 1.88 ns, respectively), and τ_3_ was found in the order of microseconds (1.32 μs). The longer lifetime value indicated a phosphorescence emission, consistent with Pt-NCN complexes.^69^ The short lifetimes may originate from physical quenching of the receptor **4** by water or oxygen (^3^O_2_) and the formation of the dimers in the excited state.^78,79^

**Figure 7:**
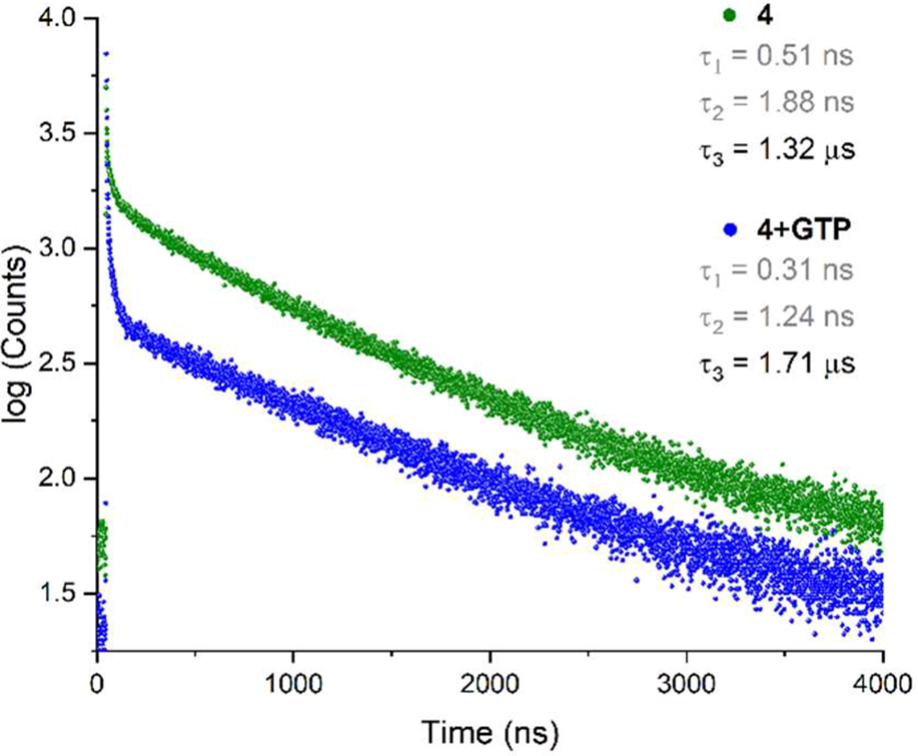
Photoluminescence decay profiles of **4** (3 μM) in the absence and presence of one equivalent of GTP in aqueous MOPS/EtOH buffer solution (7:3, 10 mM, pH = 7.4). λ_exc_ = 350 nm and λ_em_ = 500 nm.

After the addition of 1 equiv of GTP to the solution of **4**, the τ_3_ value showed a slight lifetime enhancement (τ_3_ = 1.71 μs) with respect to the lifetime of receptor **4**. Lifetime enhancement can be attributed to the formation of a more rigid complex between **4** and GTP, thereby reducing nonradiative pathways such as vibrational relaxation. Since τ_3_ did not decrease in the presence of GTP, the quenching mechanism is most likely via static quenching. The quantum yield of **4**-**GTP** was determined usingeq [Disp-formula eq2], yielding a value of Φ_f_ = 0.007, which is lower than that obtained for **4**. This significant decrease in quantum yield can be associated with emission quenching of receptor **4** by the PET mechanism.

To understand the binding mode of GTP with **4**, ^1^H and ^31^P NMR measurements were performed in D_2_O. Figure 8A shows the aromatic region of ^1^H NMR spectra of GTP and after the addition of 1–2 equiv of receptor **4**. The H_a_ signal presented a slight downfield shift (from 8.11 to 8.16 ppm), indicating an interaction of the nitrogenous base with **4** (see Figure 4D for atom labeling). This downfield shift was attributed to coordination between the Pt atom and N7 atom of GTP, deshielding the closest hydrogen atom (H_a_). It is well-known that the N7 atom of guanine is the most common binding site for the Pt-metallic center.^80^ For the H_b_ signal, an upfield shift was observed (from 5.91 to 5.86 ppm), which can be explained by shielding effects from benzimidazole units. In the case of receptor **4**, aromatic signals appeared broad and poorly resolved, indicating that receptor **4** formed aggregates, a typical feature of amphiphilic compounds in water.^45^ Nevertheless, it was possible to determine that the Pt-metallic center was the binding site to coordinate the N7 atom of GTP. In ^31^P NMR spectra (Figure 8B), GTP signals were observed at −12.84 (γ-P), −13.01 (α-P), and −22.99 ppm (β-P). Upon the addition of 2 equiv of receptor **4**, the γ-P signal presented a downfield shift to −12.50 ppm, whereas α-P and β-P signals were found at −12.66 and −22.38 ppm, respectively. These observations indicated that the triphosphate group was participating in the GTP detection, possibly through H-bonds with the tetraethylene glycol chains.

**Figure 8:**
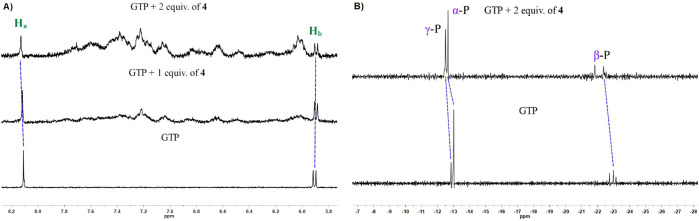
(A) ^1^H and (B) ^31^P NMR (300 and 121.5 MHz, respectively) spectra of GTP (3.82 mM) and after the addition of 1–2 equiv of **4** in D_2_O.

### Colorimetric CTP Recognition

Figure 9A shows changes in the absorption spectrum of **4** (15 μM) upon the addition of different NTPs, nucleosides, and oxyanions ([analyte]_total_ = 45 μM) in aqueous MOPS/EtOH buffer solution (7:3, 10 mM, pH = 7.4). In all cases, the presence of the analytes generated a hypochromic effect in the absorption band at 380 nm. Among all of the analytes studied, only CTP produced an absorption band at 632 nm, leading to a visible color change of the solution from colorless to green. A photograph of receptor **4** in the presence of the studied analytes is shown in Figure 9B.

**Figure 9:**
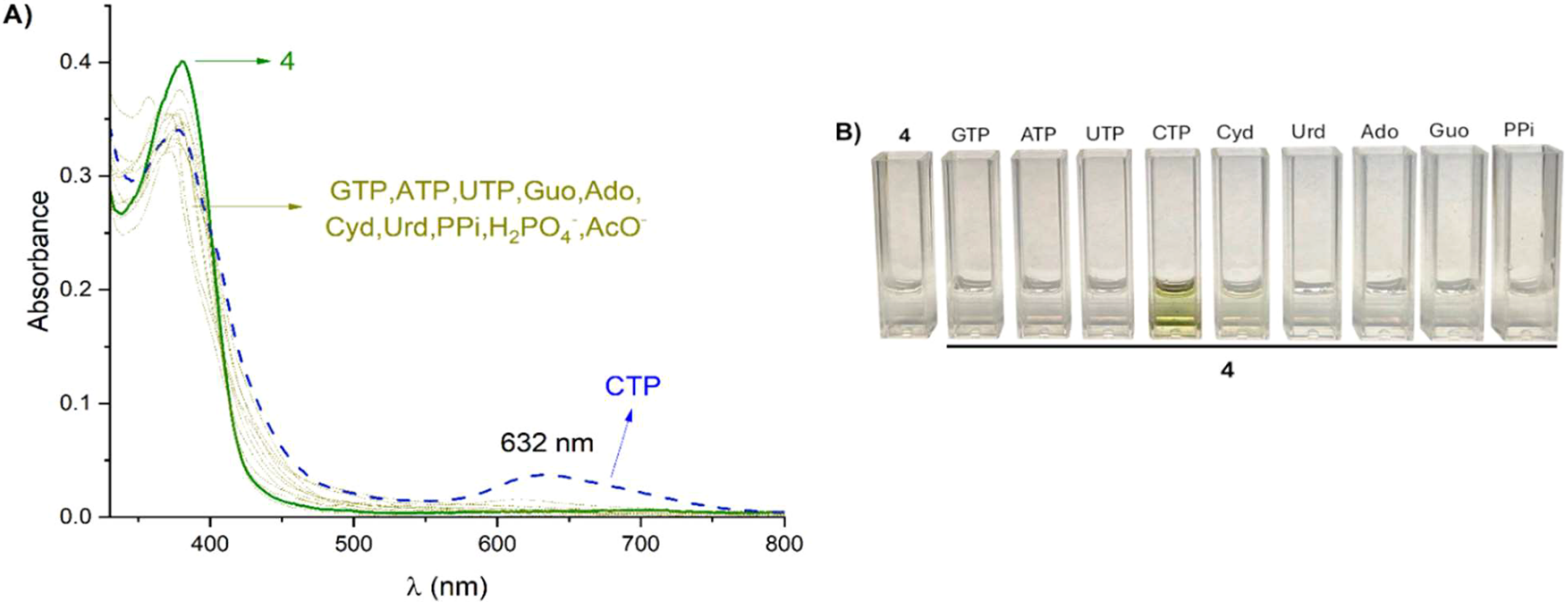
(A) Absorption spectra of receptor **4** after the addition of analytes in aqueous MOPS/EtOH buffer solution (7:3, 10 mM, pH = 7.4). (B) Photograph of solutions of receptor **4** with different analytes under natural light.

Absorption titration experiment of receptor **4** (15 μM) with CTP is depicted in Figure 10. The addition of CTP led to the appearance of a lower-energy band at 632 nm, together with a gradual decrease in the band intensity at 380 nm. Additionally, two isosbestic points at 358 and 403 nm were observed, which suggested that two species were in equilibrium, receptor **4** and **4-CTP** supramolecular complex. According to a reported Pt-NNN complex, the absorption band at ≈600 nm is typically assigned to the MMLCT [dσ*­(Pt···Pt) → π*­(terpy)] transitions caused by Pt···Pt and π–π interactions.^81^ Therefore, the colorimetric response observed in CTP detection was due to the formation of aggregates.

**Figure 10:**
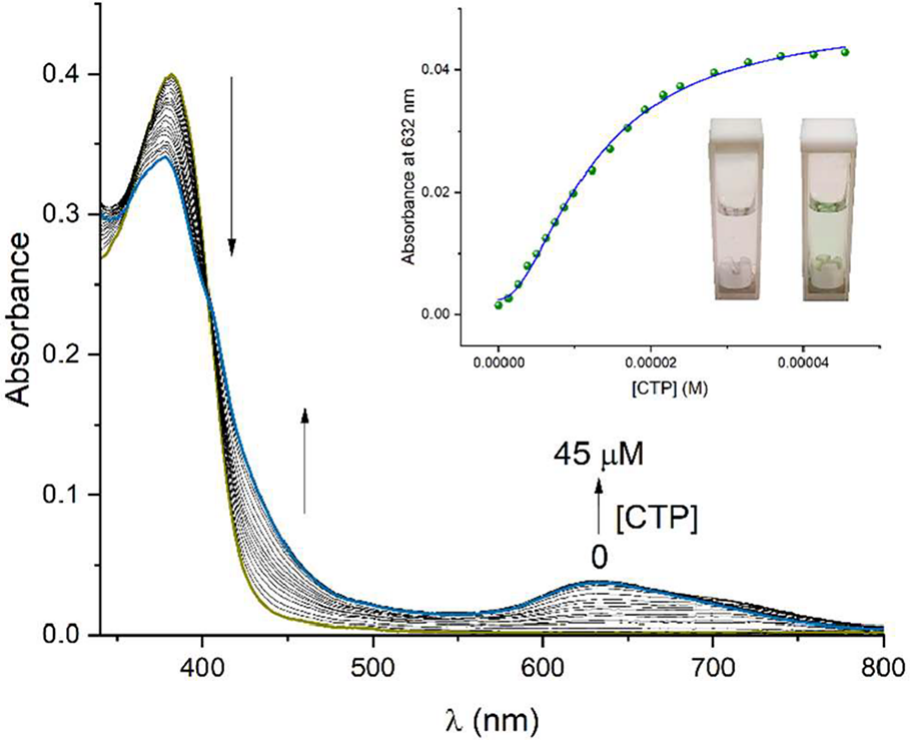
UV–vis titration of receptor **4** (15 μM) upon the addition of increasing amounts of CTP (0–45 μM) in aqueous MOPS/EtOH buffer solution (7:3, 10 mM, pH = 7.4). The inset shows a plot of the absorbance maxima at 632 nm as a function of the CTP concentration. The curve was fitted using the proposed mathematical model (see eq S3 in Supporting Information).

Plot of the absorbance maxima at 632 nm as a function of the CTP concentration showed a slight sigmoidal-like isotherm, suggesting a cooperative process (Figure 10, inset), involving the formation of a 1:1 supramolecular complex (*K*_1:1_), followed by an oligomerization process (*K*_M_). Thus, a mathematical model integrating the *K*_1:1_ and *K*_M_ constants was required. Based on the mathematical model reported by Wong and co-workers to calculate the *K*_1:1_, *K*_2:1,_ and *K*_M_ constants in a Pt-complex for PPi detection via self-assembly,^82^ we deduced a mathematical model that considers the 1:1 binding constant and the oligomerization process (pp S14 and S15, Supporting Information). The isotherm obtained from the titration of complex **4** with CTP was well fitted using this model, and the calculated values of log *K*_1:1_ and log *K*_*M*_ were 5.32 ± 0.03 and 4.55 ± 0.02, respectively.

A ratiometric plot of *A*_632 nm_/*A*_380 nm_ vs the concentration of CTP presented a good linearity (*R* ^2^ = 0.999) in the range of 1.24–9.80 μM (Figure 11A). The limit of detection was estimated to be LOD = 2.29 ± 0.02 μM. The selectivity of receptor **4** toward CTP was evaluated using an *A*_632 nm_/*A*_380 nm_ ratio for each analyte at a concentration of 45 μM. As shown in Figure 11B, only CTP induced a significant change in ratiometric absorption. Interestingly, the absence of the triphosphate group in Cyd resulted in no change in ratiometric absorption, suggesting that the triphosphate group in CTP supports aggregate formation through Pt···Pt and π–π interactions.

**Figure 11:**
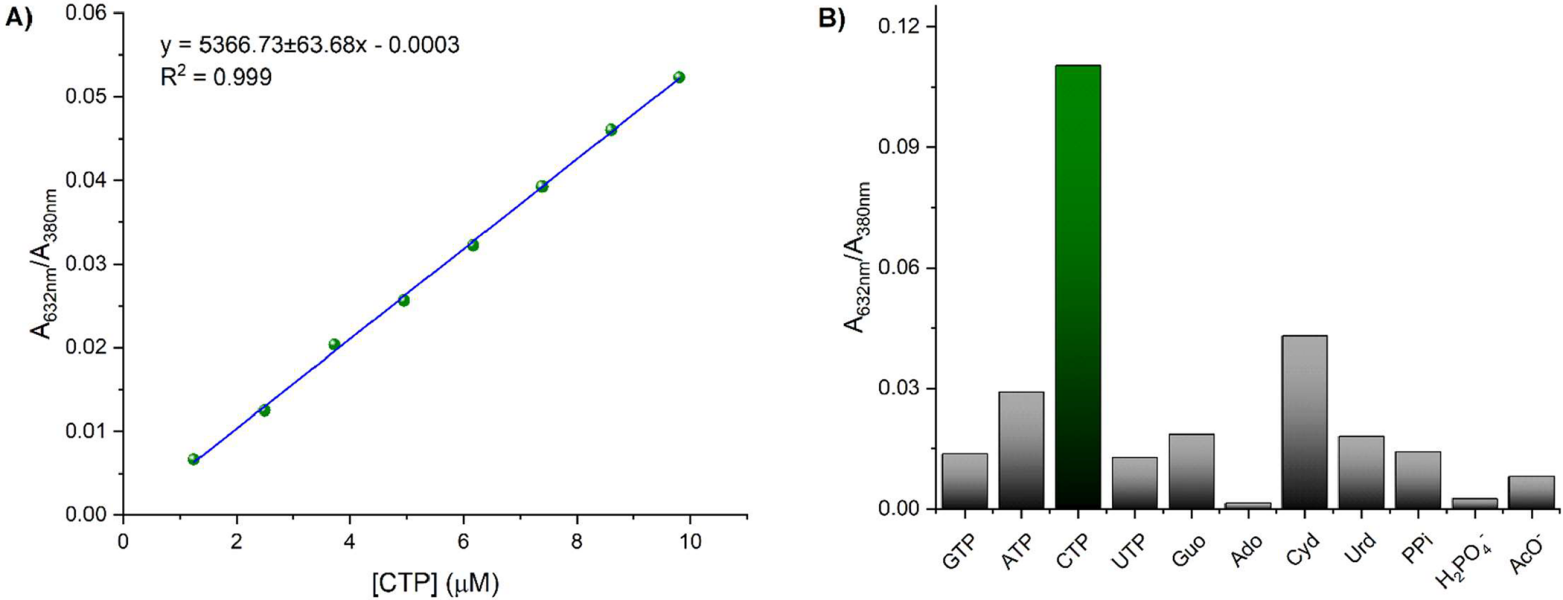
(A) Linear plot of *A*_630 nm_/*A*_380 nm_ ratio vs the concentration of CTP (1.24–9.80 μM). (B) Absorption-ratio responses of each analyte at a concentration of 45 μM.

The MALDI-TOF­(+) HRMS showed that the isotopic distribution of the species at 1378.5326 *m*/*z* corresponded to the theoretical isotopic distribution of the species [**4** + CTP + H^+^]^+,^ confirming the formation of the **4-CTP** supramolecular complex (Figure S20). Additionally, a second species at 1747.1732 *m*/*z* corresponded to the molecular weight and isotopic distribution of the dimeric species [(**4**)_2_ – Cl^–^]^+^, giving evidence of aggregates in solution.

After slow evaporation from an aqueous MOPS/EtOH buffer solution (7:3, 10 mM, pH = 7.4), luminescent microscopic fibers appeared. These fibers were observed with an optical microscope under natural and UV light, as illustrated in Figure 12A. Under UV light, these fibers have a faint green emission, which is consistent with the residual emission observed in the fluorometric titration of receptor **4** with the CTP (Figure S21). The morphology of the fiber was investigated by scanning electron microscopy (SEM), revealing that the fiber was at least 100 μm in length and exhibited a nonbranching morphology (Figure 12B). Interestingly, transmission electron microscopy coupled with energy-dispersive X-ray spectroscopy (TEM-EDS) elemental mapping at 2.5 μm showed that the fiber exhibited a homogeneous distribution of sodium, chlorine, phosphorus, and platinum atoms (Figure 12C), confirming that the fiber was formed by the **4-CTP** supramolecular complex.

**Figure 12:**
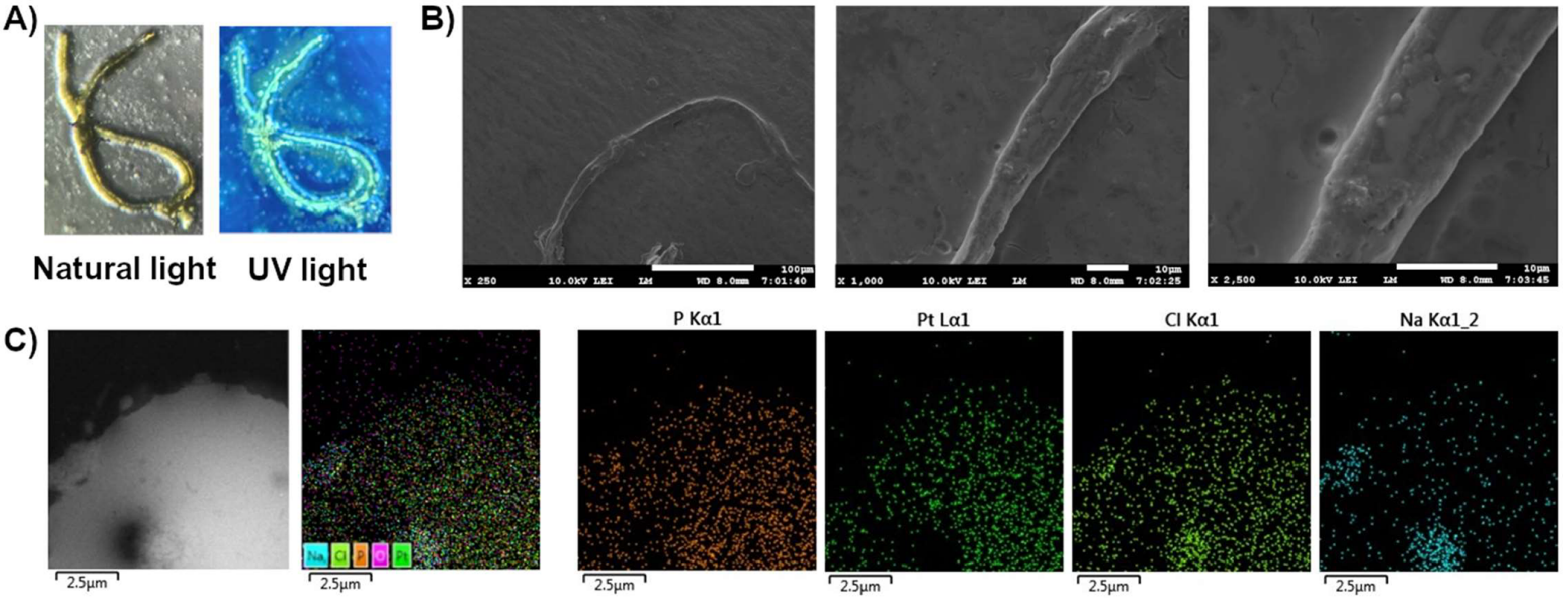
(A) Fiber-like aggregate of **4-CTP** observed by microscope under natural and UV light. (B) SEM images of the fiber. (C) TEM-EDS elemental mapping of the fiber at 2.5 μm.

To gain insight into the binding mode of the CTP and the formation of aggregates, ^1^H and ^31^P NMR measurements were carried out in D_2_O. Figure 13A shows ^1^H NMR spectra of the aromatic region of CTP and the addition of 1–2 equiv of **4**. The H_a_ signal displayed a pronounced downfield shift from 8.07 to 8.23 ppm (see Figure 4D for atom labels). Similarly, the H_b_ signal shifted downfield from 6.21 to 6.33 ppm. These shifts were attributed to the coordination of the Pt-metallic center with the N3 atom of CTP, which led to deshielding of H_a_ and H_b_ atoms. In contrast, the H_c_ signal showed a slight upfield shift from 5.97 to 5.94 ppm, which can be attributed to shielding effects by aggregation. Thus, ^1^H NMR measurements confirmed the coordination of the Pt atom to the N3 atom of CTP. In ^31^P NMR spectra (Figure 13B), three signals at −10.81 (γ-P), −11.44 (α-P), and −23.15 ppm (β-P) were observed for CTP. Upon the addition of 2 equiv of **4**, γ-P and α-P signals showed an upfield shift to −10.98 and −11.50 ppm, respectively, while the β-P signal remained at −23.12 ppm. Additionally, the phosphate signals became broader and less visible. This behavior suggested the formation of supramolecular aggregates in solution. In such systems, molecular mobility is reduced, and spin–spin relaxation is enhanced, leading to broad and less intense signals.^82^ The slight chemical shift observed suggested that the triphosphate group experienced changes in its chemical environment, likely due to noncovalent interactions such as hydrogen bonding with the receptor. To gain further insights into the recognition mode, DFT calculations were carried out.

**Figure 13:**
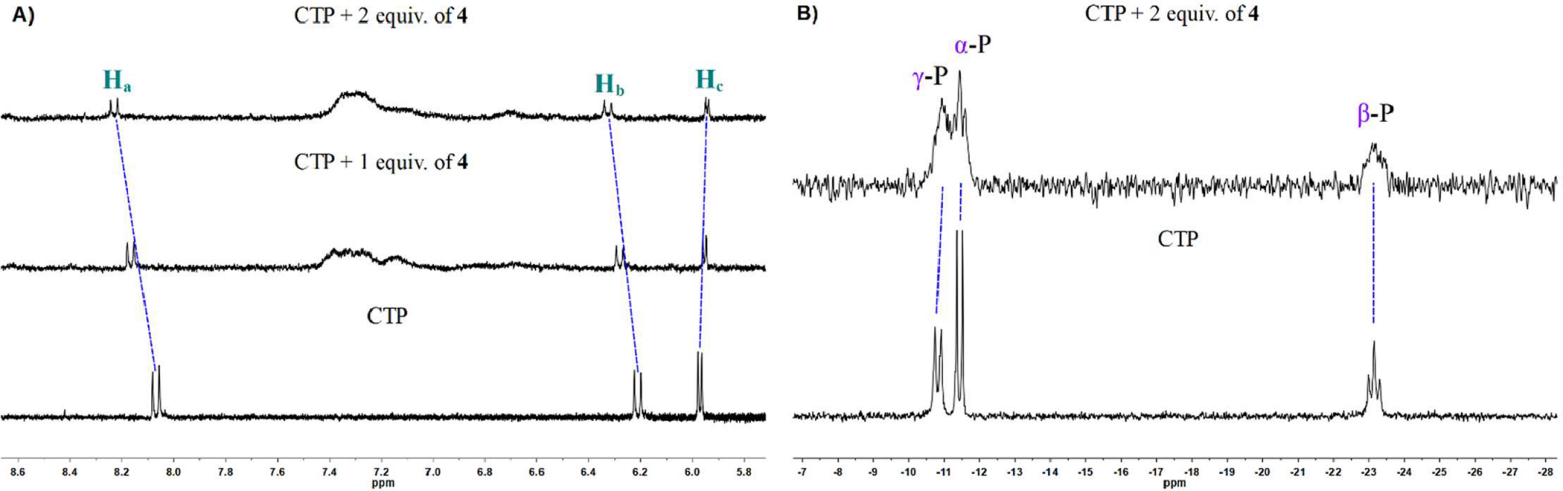
(A) ^1^H and (B) ^31^P NMR (300 and 121.5 MHz, respectively) spectra of CTP (3.80 mM) and the addition of 1–2 equiv of **4** in D_2_O.

### DFT Calculations

The geometry optimizations of the 1:1 ligand-nucleotide complexes **4-GTP** and **4-CTP** were performed at the B3LYP DFT-D3­(BJ) 6-31G­(d,p)/LANL2DZ/MWB60 level of theory. Molecular geometries of **4-GTP** and **4-CTP** are depicted in Figure 14A,B, respectively. The complexes were modeled with three Na atoms to stabilize the negative triphosphate charges. Two Na centers were localized between TEG chains of the ligand and phosphate groups of the nucleotides. The platinum center served as the anchoring site for the nucleotide residue at the N atom.

**Figure 14:**
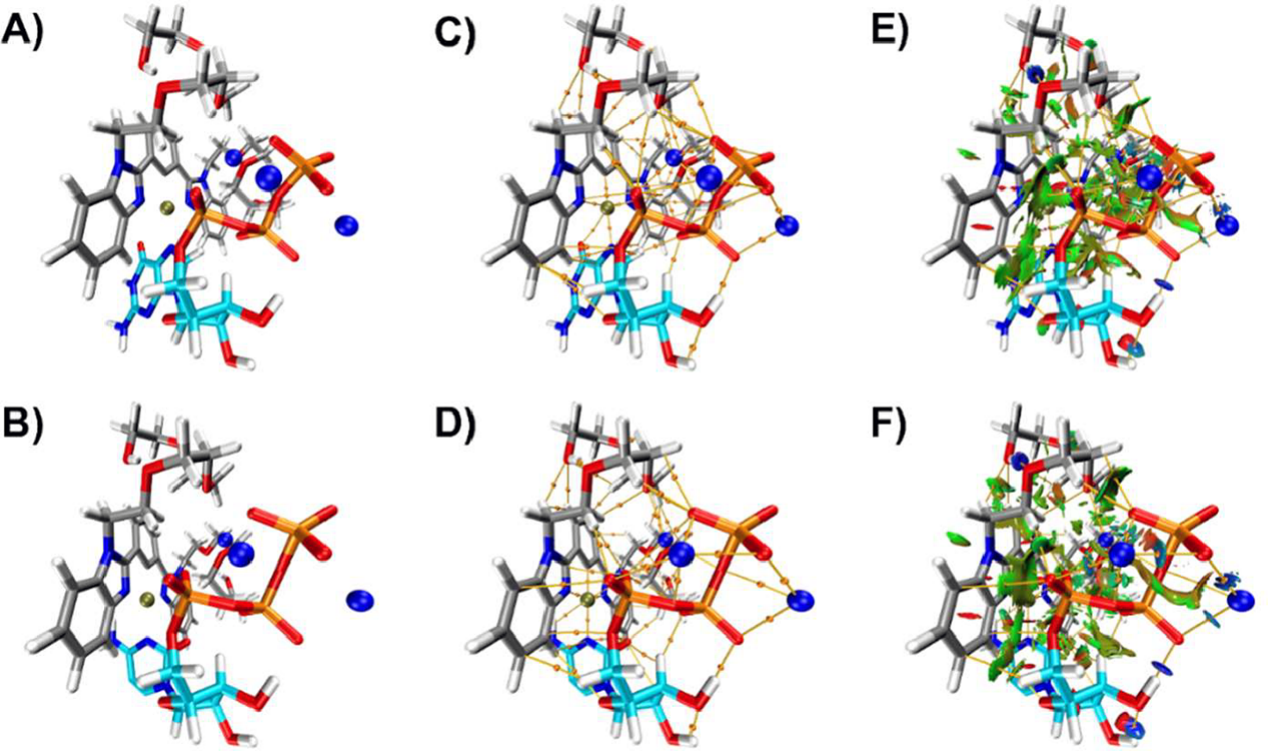
(A) and (B) Molecular graphs obtained from geometry optimization at B3LYP DFT-D3­(BJ) 6-31G­(d,p)/LANL2DZ/MWB60 level of theory; (C) and (D) AIM molecular geometries with BCPs and BPs of noncovalent interactions; (E) and (F) NCI isosurfaces together with QTAIM CPs and BPs. Upper row corresponds to **4-GTP** and lower row corresponds to **4-CTP**. AIM and NCI analysis were performed using Multiwfn.^60^

Molecular graphs were obtained from topology analysis of electron density using QTAIM; bond critical points (BCP) and connecting bond paths (BP) are depicted in Figure 14C,D, for **4-GTP** and **4-CTP**, respectively. The triphosphate moiety interacted with the TEG chains of the ligand and sodium atoms. Hydrogen bonds were formed between phosphate oxygen atoms and the −OH– groups of TEG chains. Three BCPs corresponding to intermolecular interactions of the Pt center with a benzene C atom, Pt–C, and two benzimidazole N atoms of the ligand, Pt–N, described the coordination of Pt with the ligand. In both complexes, a BCP corresponding to intermolecular contact Pt···N between the N heteroatom of nucleotide and Pt atom revealed the main anchoring site for nucleotides to the ligand.

Contour line maps of the Laplacian of the electron density revealed the closed-shell interaction nature of Pt bonds in **4-GTP** and **4-CTP**, Figure 15A,B, respectively. These contacts were described as noncovalent ionic bonds since the positive sign of the Laplacian value at the BCP and positive contour lines of Pt were observed. The values of electron density ρ­(*r*), Laplacian of the density (∇^2^ρ­(*r*)), Hessian eigenvalues (λ_1_, λ_2_, and λ_3_), and ellipticity (ε) of BCPs comprising contacts with the Pt center are presented in Table 2.

**Figure 15:**
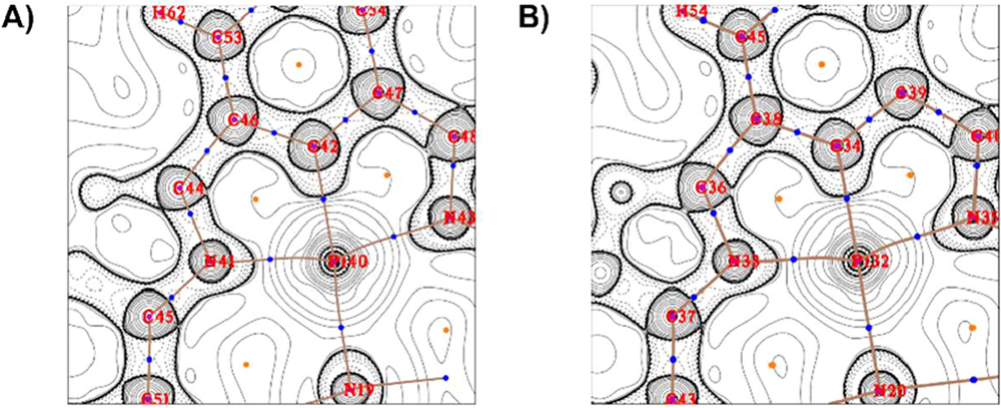
Plot lines of Laplacian of **4-GTP** and **4-CTP**, (A) and (B), respectively. The 2D plots were obtained using Multiwfn’s built-in QTAIM plotting routine.^60^

**Table 2:** Bond Critical Point (BCP) Information of **4-GTP** and **4-CTP** Complexes

					Hessian eigenvalues			
complex^a^	BCP	type	ρ(*r*)	∇^2^ρ(*r*)	λ_1_	λ_2_	λ_3_	ε
(a) **4-GTP**	Pt40–C42	(3,–1)	0.1695	0.1837	–0.2089	–0.1991	0.5917	0.0489
	Pt40–N19	(3,–1)	0.0841	0.3798	–0.0831	–0.0654	0.5282	0.2708
	Pt40–N41	(3,–1)	0.1110	0.4399	–0.1265	–0.1175	0.6839	0.0768
	Pt40–N43	(3,–1)	0.1125	0.4511	–0.1307	–0.1189	0.7008	0.0988
(b) **4-CTP**	Pt32–C34	(3,–1)	0.1685	0.1608	–0.2103	–0.2003	0.5714	0.0498
	Pt32–N20	(3,–1)	0.0790	0.3555	–0.0720	–0.0560	0.4835	0.2870
	Pt32–N33	(3,–1)	0.1142	0.4425	–0.1312	–0.1219	0.6956	0.0760
	Pt32–N35	(3,–1)	0.1119	0.4446	–0.1288	–0.1173	0.6907	0.0984

a See Figure 15 for atom numbering.

The visual analysis of the reduced density gradient by NCI complemented the QTAIM analysis. The isosurfaces illustrated the regions of intermolecular interactions with a color code corresponding to the sign­(λ_2_)­ρ. The isosurfaces are displayed along with the BCPs and BPs of molecular geometries of **4-GTP** and **4-CTP**, in Figure 14E,F, respectively. In both complexes, weak noncovalent interactions appeared as extended green colored isosurfaces in the region between TEG chains and triphosphate moiety. Stronger interactions were observed as blue isosurfaces, corresponding to intramolecular OH···O, PO···H, and PO···Na interactions.

The complexation energies of **4-GTP** and **4-CTP** were corrected for BSSE, which is an artificial stabilization of weakly bound molecular complexes. The corrected counterpoise energies of the **4-GTP** and **4-CTP** complexes in the gas phase were −147.0 and −142.1 kcal/mol, respectively, **4-GTP** being 4.9 kcal/mol more stable than the **4-CTP** complex. The calculated uncorrected (raw) complexation energies were −169.4 and −164.7 kcal/mol. BSSE energies accounted for 22.4 and 22.5 kcal/mol for **4-GTP** and **4-CTP**, respectively.

### Comparative Table of Receptors for GTP and CTP

Recently reported chemical sensors used for the detection and quantification of GTP and CTP in aqueous media are listed in Table 3. The listed receptors are either coordination complexes or organic molecules, with binding affinities ranging from log *K* = 3.92 to 6.05 and log *K* = 4.07 to 4.43 for GTP and CTP, respectively. In this work, we report a dual-mode receptor based on a Pt-NCN complex (**4**) for luminescent detection of GTP and chromogenic detection of CTP, with binding constants of log *K* = 6.85 and log *K* = 5.32, respectively. The main interactions involved in the recognition of GTP and CTP were a coordination bond between the Pt atom and the N atom of the nitrogenous base, as well as hydrogen bonds between TEG chains and the triphosphate group. Additionally, CTP detection involved the formation of supramolecular aggregates, assisted by Pt···Pt and π–π stacking interactions.

**Table 3:** Recent Chemosensors for the Detection and Quantification of GTP and CTP in Aqueous Media

chemosensor	method	LOD	binding constant	analyte	reference
dinuclear Zn(II)-dipicolylamine complex	fluorescence turn off	9.2 μM	log *K*_1:1_ = 6.05	GTP	^21^
tris-naphthoimidazolium compound	fluorescence turn off	^a^	log *K*_1:1_ = 4.81	GTP	^27^
benzimidazolium-based cyclophane	fluorescence turn off	26.7 μM	log *K*_1:2_ = 4.69	GTP	^23^
imidazolium-functionalized squaraines	colorimetric and fluorescence response	5.4 ppb	^a^	GTP	^25^
cationic tentacle porphyrin	fluorescence turn on	2.3 μM	log *K*_1:1_ = 3.92	GTP	^26^
tetraphenylene-based tetraimidazolium cyclophane	fluorescence turn on	16 μM	log *K*_1:1_ = 5.19	GTP	^22^
bisquinolinium compound	fluorescence turn off	^a^	log *K*_1:1_ = 4.70	GTP	^24^
spider-like Schiff-base receptor	fluorescence turn off	8.3 μM	log *K*_1:1_ = 4.07	CTP	^28^
naphthalimide-piperazine macrocycle	fluorescence turn on	250 nM	log *K*_1:1_ = 4.43	CTP	^29^
Pt(II)-NCN complex (**4**)	fluorescence turn off	1.28 μM	log *K*_1:1_ = 6.85	GTP	this work
	colorimetric response	2.29 μM	log *K*_1:1_ = 5.32	CTP	

a Data not available.

## Conclusions

We developed a water-soluble Pt-NCN complex (**4**) based on a 1,3-bis­(benzimidazole)­benzene derivative bearing two tetraethylene glycol chains for the recognition of nucleotides, nucleosides, and oxyanions in aqueous media at pH = 7.4. Receptor **4** displayed the ability to dual detect GTP and CTP at micromolar levels in a 1:1 binding mode. Receptor **4** exhibited its strongest binding affinity toward GTP (log *K*_1:1_ = 6.85 ± 0.01) via turn-off response with a limit of detection of 1.28 μM. On the other hand, CTP induced a color change of receptor **4** solution, from colorless to green, associated with the formation of fiber-like aggregates driven by Pt···Pt and π–π interactions. The apparent binding constant (log *K*_1:1_ = 5.32 ± 0.03) and oligomerization constant (log *K*_*M*_ = 4.55 ± 0.02) were determined using a proposed mathematical model. The determined limit of detection value for CTP was 2.29 μM. The experimental investigation of the sensing mode of GTP and CTP indicated that receptor **4** recognized these nucleotides by two binding sites: the coordination of the Pt atom with N7 or N3 atoms of GTP and CTP, respectively, and hydrogen bonds between TEG chains and the triphosphate group. DFT calculations supported the experimental observation, determined by noncovalent interactions depicted as NCI isosurfaces and BCPs related to H···OP and Pt–N intermolecular contacts. These results further highlight the utilization of the Pt-NCN complex as a dual luminescent and chromogenic chemosensor for the detection of GTP and CTP in aqueous media.

## Supplementary Material





## Notes

Reagents, solvents and equipment. ^1^H, ^13^C NMR spectra of **1**-**4**, ESI-MS spectra of compounds. IR-ATR spectra of compounds. Crystallographic description. Spectroscopic experiments of complex **4**. Proposed mathematical model to calculate the binding constant *K*_1:1_ and oligomerization process *K*_M_. DFT Cartesian coordinates of optimized complexes and AIM and NCI molecular graphs (PDF)
All geometries (XYZ)
