# Supplementary material for: A Dual Luminescent and Chromogenic Pt-NCN Complex for the Detection of GTP and CTP in Aqueous Media
Source: Inorg Chem. 2026 Jan 5;65(2):1436–51. doi: 10.1021/acs.inorgchem.5c04987 (PMC12820956; doi:10.1021/acs.inorgchem.5c04987)
Supplement: Supplementary file 1 [file ic5c04987_si_001.pdf]

A dual luminescent and chromogenic Pt-NCN complex for the detection of GTP and CTP in aqueous media.

Josue Valdes-García,[a]* Mireille Vonlanthen,[a] Fabián Cuétara-Guadarrama,[a] David Garcia-Bassoco,[a] Hector Luis Valdés-Negrín,[a] Arturo López-Pérez,[a] Ernesto Rivera,[a]* Simón Hernández-Ortega,[b] and Alejandro Dorazco-González[b]*

[a]Instituto de Investigaciones en Materiales, Universidad Nacional Autónoma de México. Circuito Exterior, Ciudad Universitaria, C.P. 04510, CDMX, México

[b]Instituto de Química, Universidad Nacional Autónoma de México. Circuito Exterior, Ciudad Universitaria, C.P. 04510, CDMX, México

Corresponding authors: J.V.G.: valdesjosue94@comunidad.unam.mx, E.R.: riverage@unam.mx and A.D.G.: adg@unam.mx

Supporting Information

Reagents, solvents and equipment.

Figure S1. ^{1}H NMR (300 MHz, 298 K, DMSO-d_6) spectrum of 1,3-bis(1H-benzo[*d*]imidazol-2-yl)benzene, **1**.

Figure S2. ^{1}H NMR (300 MHz, 298 K, $CDCl_3$) spectrum of **TEG-Br** (It contains 9.76% of triphenylphosphine).

Figure S3. ^{1}H NMR (300 MHz, 298 K, $CDCl_3$) spectrum of **2**.

Figure S4. ^{13}C NMR (75.5 MHz, 298 K, $CDCl_3$) spectrum of **2**.

Figure S5. Positive scan ESI-MS spectrum of **2** in ethyl acetate.

Figure S6. ATR-IR spectrum of **2**.

Figure S7. ^{1}H NMR (300 MHz, 298 K, $CDCl_3$) spectrum of **3**.

Figure S8. ^{13}C NMR (75.5 MHz, 298 K, $CDCl_3$) spectrum of **3**.

Figure S9. Positive scan ESI-MS spectrum of **3** in ethyl acetate.

Figure S10. ATR-IR spectrum of **3**.

Figure S11. ^{1}H NMR (300 MHz, 298 K, $CDCl_3$) spectrum of **4**.

Figure S12. ^{13}C NMR (75.5 MHz, 298 K, $CDCl_3$) spectrum of **4**.

Figure S13. Positive scan ESI-MS spectrum of **4** in acetonitrile.

Figure S14. ATR-IR spectrum of **4**.

Figure S15. **A**). Dimers chain along the *b*-axis formed by π-π stacking interaction (3.67 Å). **B**) 1D chain along the *c*-axis induced by dimeric $R_2^2(10)$ homo-synthon. **C**) 1D chain via C-H···Cl interaction along the *a*-axis. Only the relevant hydrogen atoms are shown for clarity.

Figure S16. 2D-fingerprint plots of the complex **4** showing the contributions of reciprocal contacts to crystal packing.

Figure S17. Absorption (15 μM) and emission (3 μM, λ_{exc}= 380 nm) spectra of **4** in aqueous MOPS/EtOH buffer solution (7:3, 10 mM, pH = 7.4).

Figure S18. Job's plot between **4** (3 μM, λ_{exc}= 380 nm) and GTP in aqueous MOPS/EtOH buffer solution (7:3, 10 mM, pH = 7.4). Line-line intersection is $\chi \approx 0.5$ confirming the 1:1 binding model.

Figure S19. Species at 1425.3347 m/z found in the HRMS-ESI(+) spectrum of the resulting solution from UV-Vis titration between receptor **4** and GTP, and the theoretical isotope distribution of the [**4** – Cl^- + GTP – $2H^+$ + $2Na^+$]$^+$.

Figure S20. Species at **A**) 1378.5326 and **B**) 1747.1732 *m/z* found in the MALDI-TOF HRMS spectrum of the resulting solution from UV-vis titration between receptor **4** and CTP, and the theoretical isotopic distribution of the [**4** + CTP + H^+]$^+$ and [(**4**)$_2$ – Cl^-]$^+$.

Figure S21. Changes in the emission spectra (λ_{exc} = 380 nm) of receptor **4** (3 μM) upon the addition of CTP (0 – 6 μM) in aqueous MOPS/EtOH buffer solution (7:3, 10 mM, pH = 7.4). The curve was fitted using equation (2).

Table S1. Crystallographic data for **4**.

Table S2. Selected bond distances (Å) and angles (°) around Pt atoms in **4**.

Table S3. Hydrogen bonds for **4** [Å and °].

Table S4. Absorption and emission maxima (nm) of **4** in aqueous MOPS/EtOH buffer solution (7:3, 10 mM, pH = 7.4).

Mathematical Model to calculate the binding constant $K_{1:1}$ an oligomerization process K_M.

Cartesian coordinates of the geometry of complex **4-GTP**.

Cartesian coordinates of the geometry of complex **4-CTP**.

Figure S21. A, B) Molecular graphs obtained from geometry optimization at B3LYP DFT-D3(BJ) 6-31G(d,p)/LANL2DZ/MWB60 level of theory.

Figure S22. Plots of contour lines of the Laplacian of electron density for the 1:1 GTP–Pt-NCN complex, **4-GTP**.

Figure S23. Plots of contour lines of the Laplacian of electron density for the 1:1 CTP–Pt-NCN complex, **4-CTP**.

Reagents, solvents and equipment

All reagents were used directly as obtained commercially from Sigma-Aldrich without further purification: orthophenylenediamine (>99.5%), isophthalic acid (>99%), $NaHCO_3$ (>99.5%), tetraethylene glycol (>99%), tetrabromomethane (>99%) , triphenylphosphine (>95%), Cs_2CO_3 (>99%), $K_2[PtCl_4]$ (>98%), KOH (>99%), GTP (>90%), ATP (>97%), UTP (>96%), CTP (>95%), adenosine (>99%), guanosine (>97%), uridine (>99%), cytidine (99%), sodium acetate (>99%), sodium pyrophosphate decahydrate (>99%), sodium phosphate monobasic (99%) and MOPS (99.5%). Solvents: Orthophosphoric acid (85% wt.% in H_2O), distilled water, acetonitrile (99.9%), ethanol (>99.5%, HPLC), dichloromethane (>99.8%, HPLC), ethyl acetate (>99.5%, ACS reagent), hexane (>98%), dry *N,N*-dimethylformamide (>99.8%), methanol (99.8%, ACS reagent), acetic acid (>99.7) and chloroform (>99.8%, ACS reagent).

UV-vis spectra were recorded on an Agilent Cary 100 UV-VIS spectrophotometer. Fluorescence spectra were recorded on an Agilent Cary Eclipse spectrophotometer. ^{1}H and ^{13}C NMR spectra of the characterization were recorded on a Bruker Advance DPX 300 spectrometer at 300 MHz. ^{1}H and ^{31}P NMR spectra of the titration experiments were recorded on a Bruker Advance DPX 300 spectrometer at 300 MHz. MS-ESI positive scan spectra were recorded on an Agilent Tech 6530BA Q-TOF LC/MS spectrometer. MALDI-TOF MS positive scan spectrum was recorded on a Bruker Microflex with MALDI-TOF. IR-ATR spectra were recorded on a Nicolet^{TM} iS50 FTIR spectrometer. Elemental analyses were performed using a Thermo Scientific Flash 2000 instrument at 950°C. SEM and TEM-EDS images were acquired using a JEOL 7600F Scanning Electron Microscope and JEOL ARM200F Transmission Electron Microscope, respectively. Time-resolved fluorescence decays were acquired using a Horiba Jobin Yvon IBH Ltd. Time-resolved fluorometer equipped with IBH 340 nm NanoLED. The samples were excited at 340 nm and fluorescence decays were acquired at 500 nm. The instrument response function was determined with a Ludox solution. The decays were fitted using the DAS6 decay analysis software from Horiba Scientific.

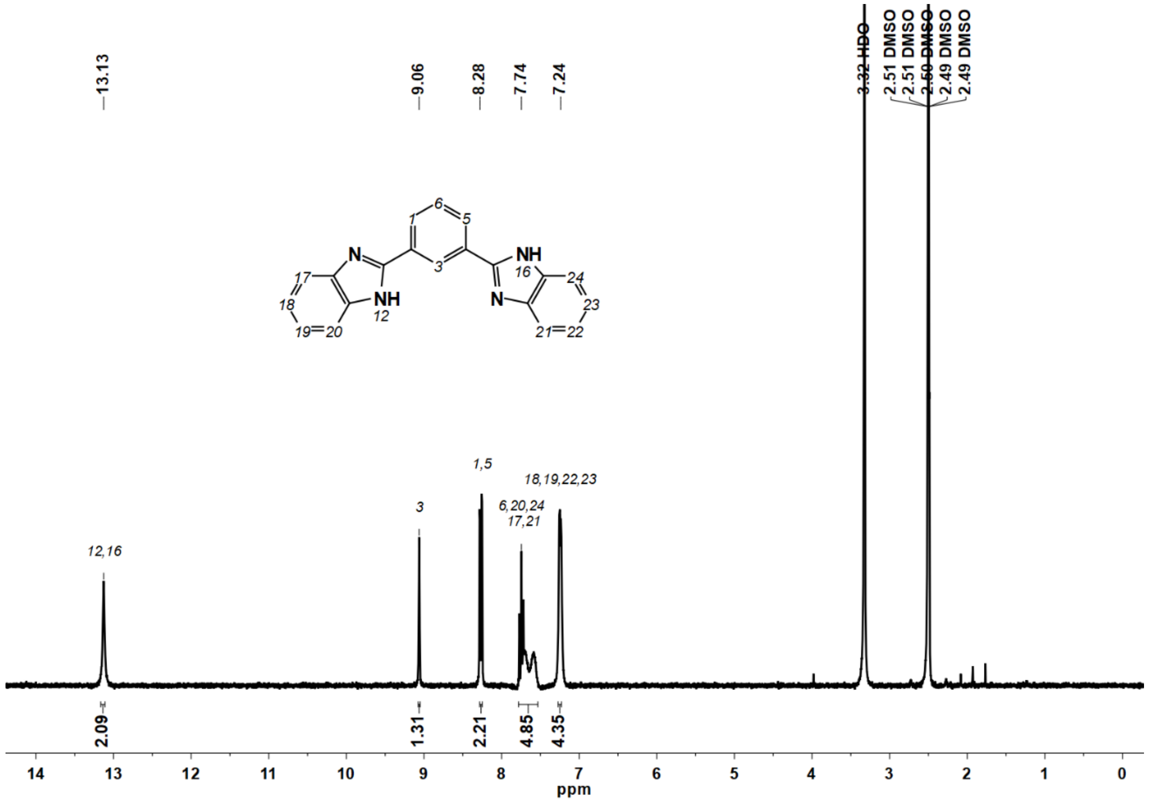

Figure S1. ^{1}H NMR (300 MHz, 298 K, DMSO-d_6) spectrum of 1,3-bis(1H-benzo[*d*]imidazol-2-yl)benzene, **1**.

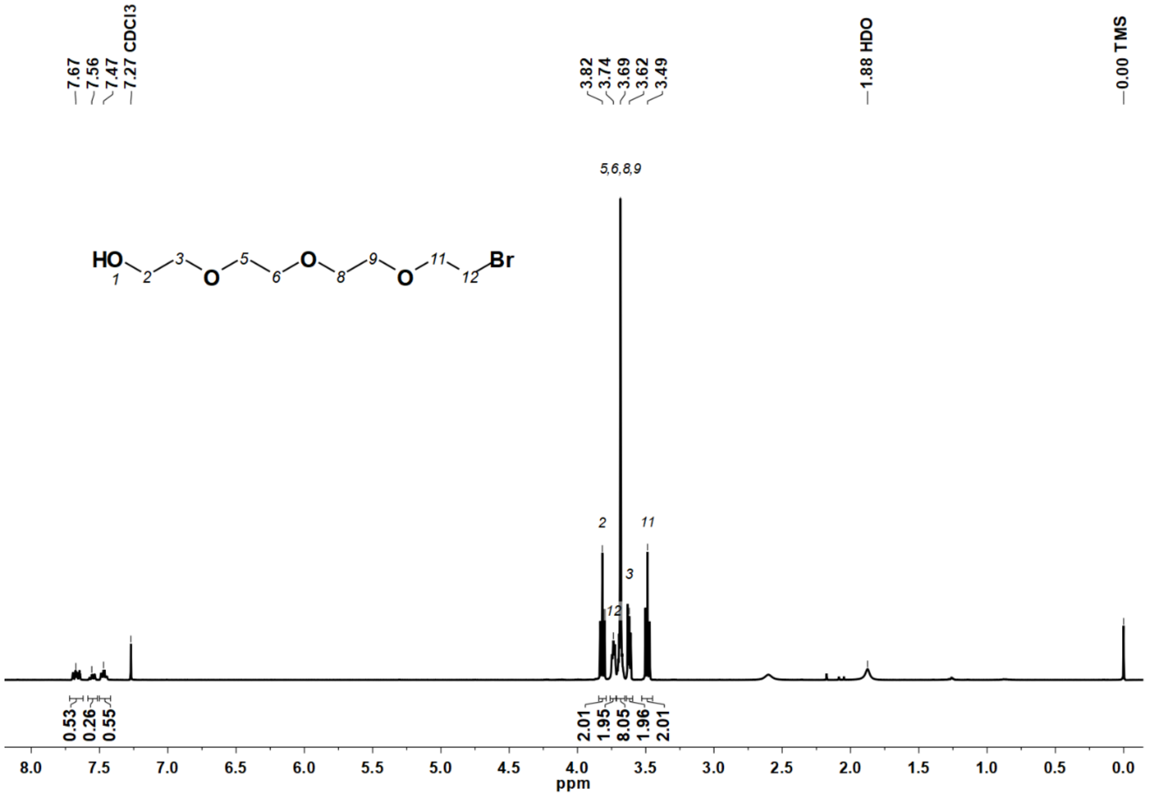

Figure S2. ^{1}H NMR (300 MHz, 298 K, $CDCl_3$) spectrum of **TEG-Br** (It contains 9.76% of triphenylphosphine).

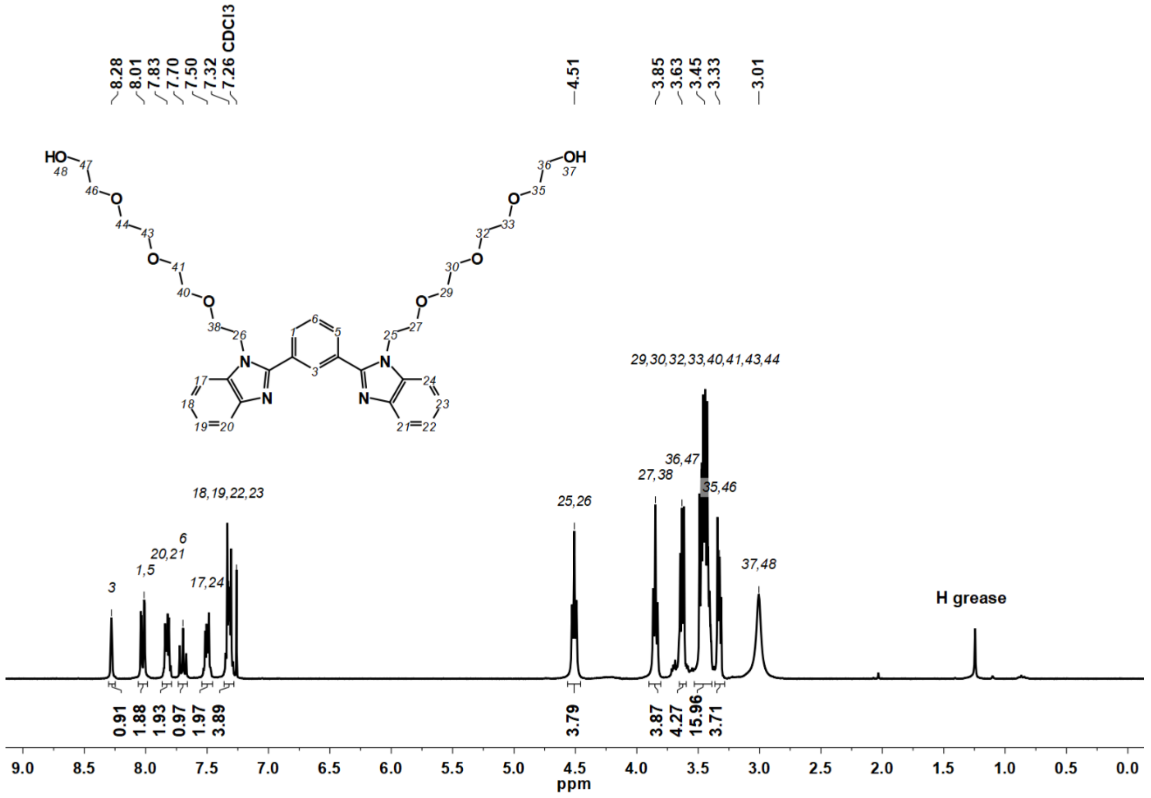

Figure S3. ^{1}H NMR (300 MHz, 298 K, $CDCl_3$) spectrum of **2**.

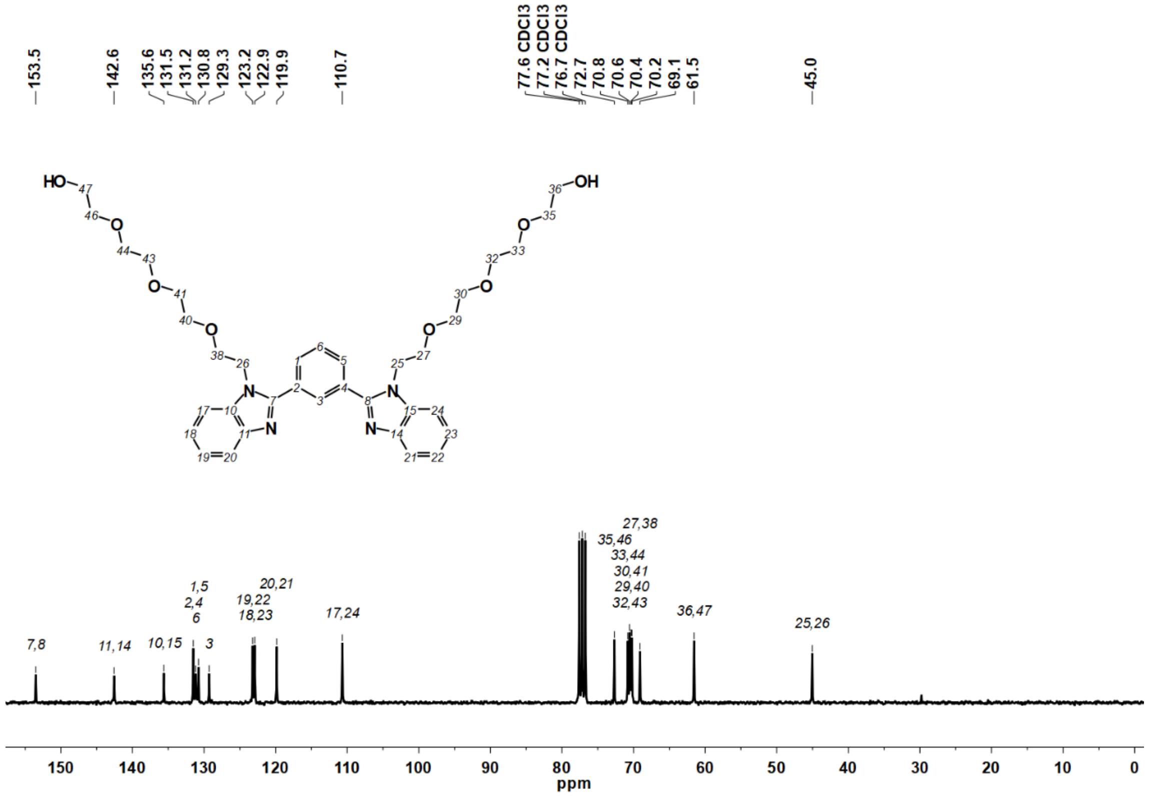

Figure S4. ^{13}C NMR (75.5 MHz, 298 K, $CDCl_3$) spectrum of **2**.

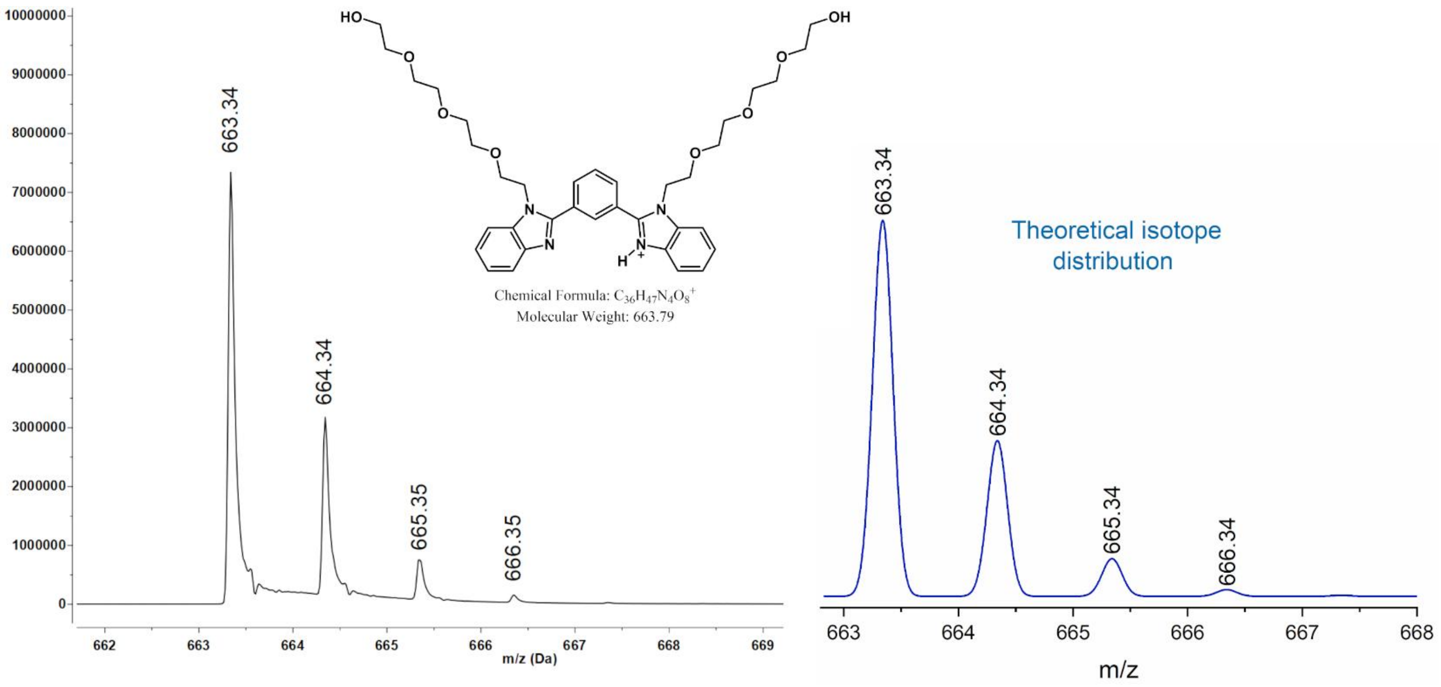

Figure S5. Positive scan ESI-MS spectrum of **2** in ethyl acetate.

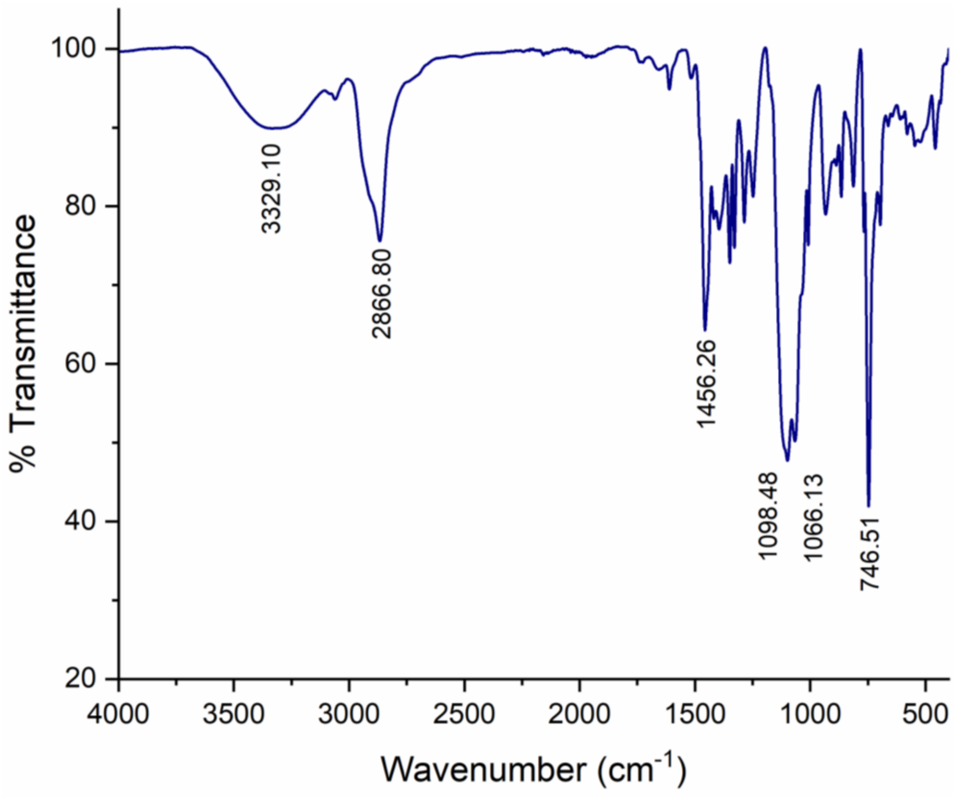

Figure S6. ATR-IR spectrum of **2**.

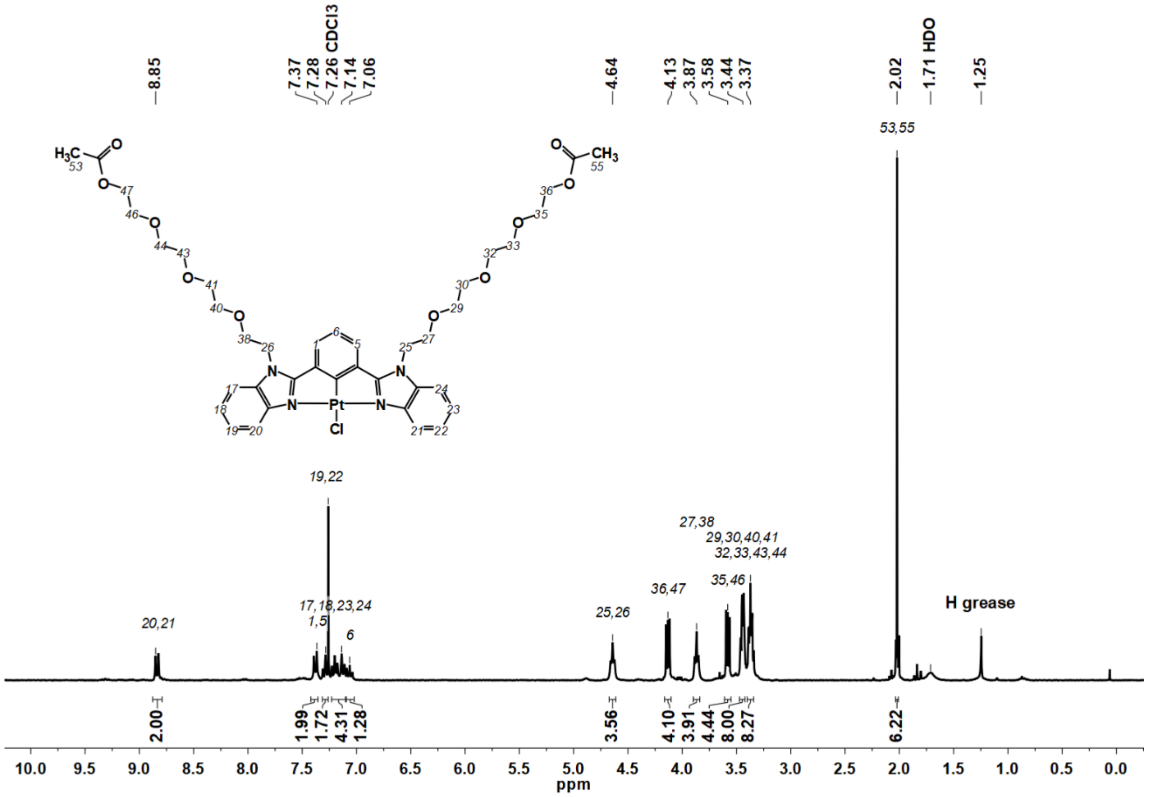

Figure S7. ^{1}H NMR (300 MHz, 298 K, $CDCl_3$) spectrum of **3**.

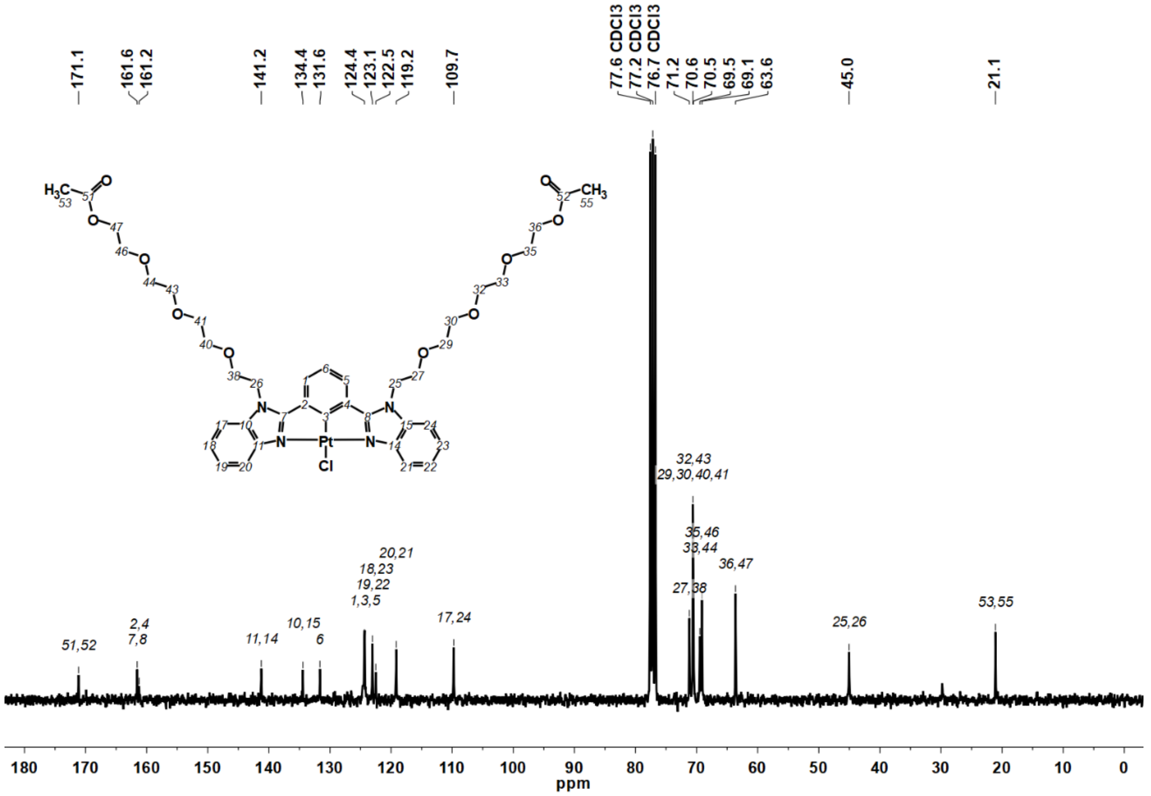

Figure S8. ^{13}C NMR (75.5 MHz, 298 K, $CDCl_3$) spectrum of **3**.

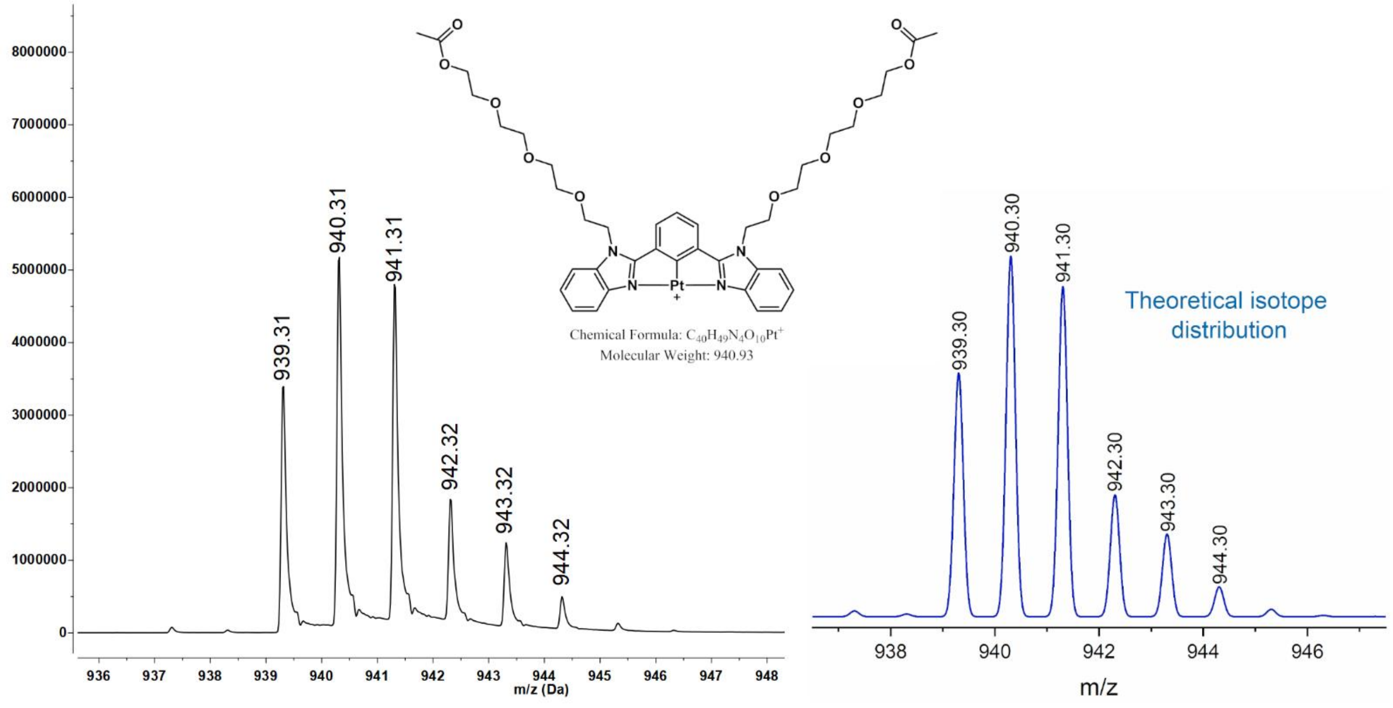

Figure S9. Positive scan ESI-MS spectrum of **3** in ethyl acetate.

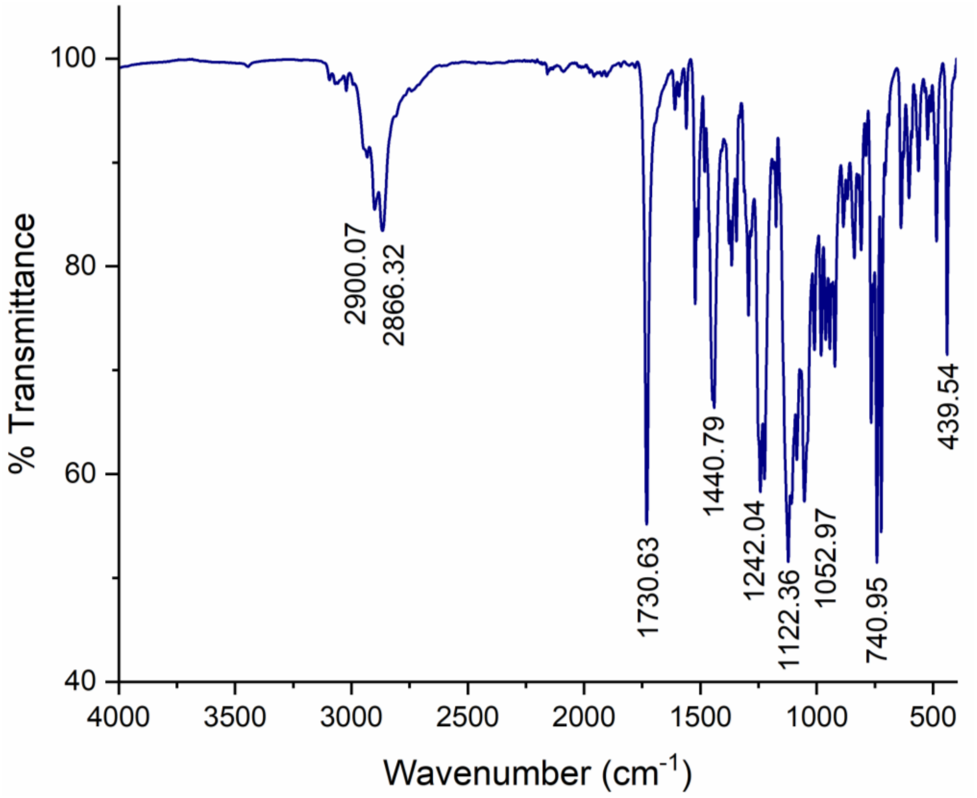

Figure S10. ATR-IR spectrum of **3**.

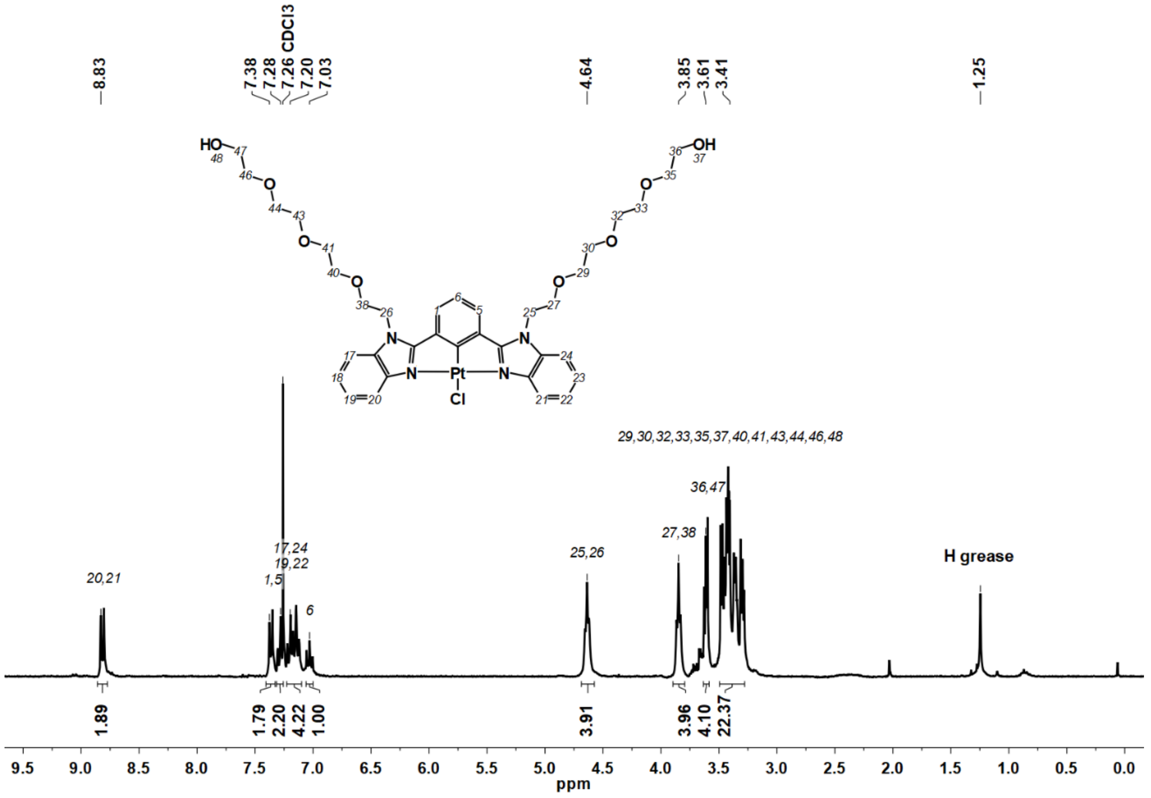

Figure S11. ^{1}H NMR (300 MHz, 298 K, $CDCl_3$) spectrum of **4**.

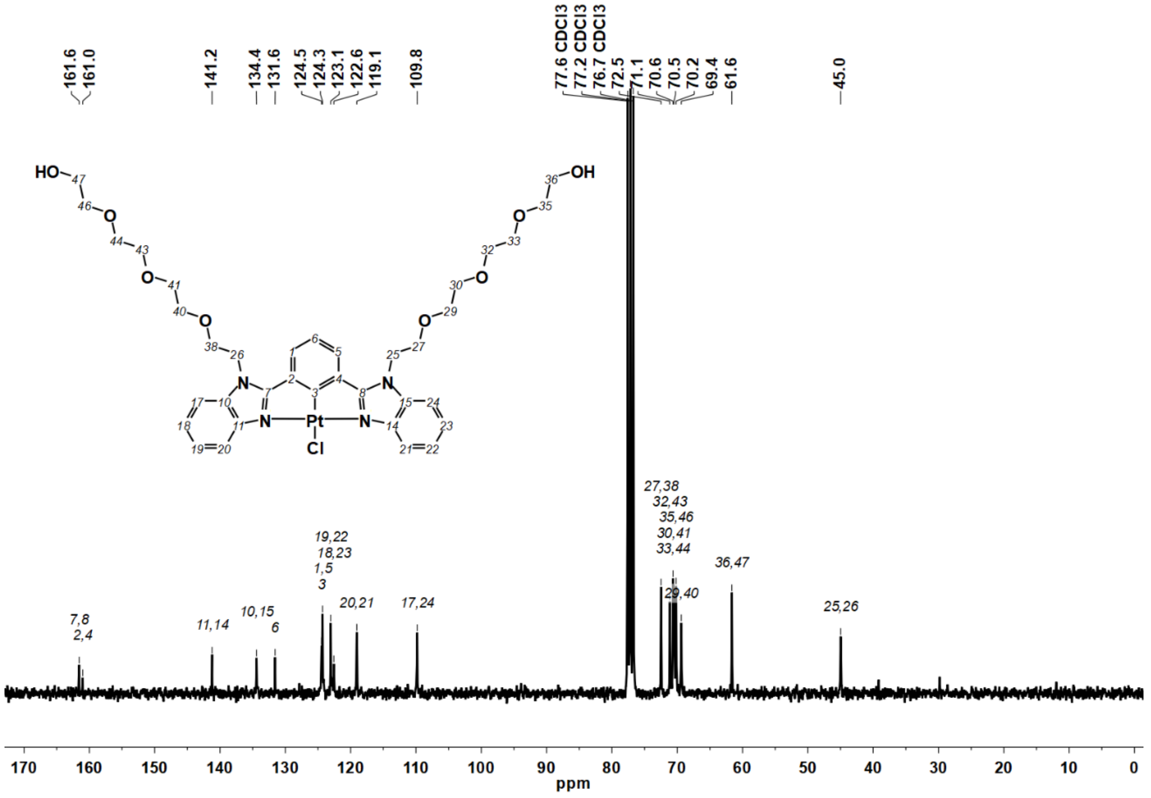

Figure S12. ^{13}C NMR (75.5 MHz, 298 K, $CDCl_3$) spectrum of **4**.

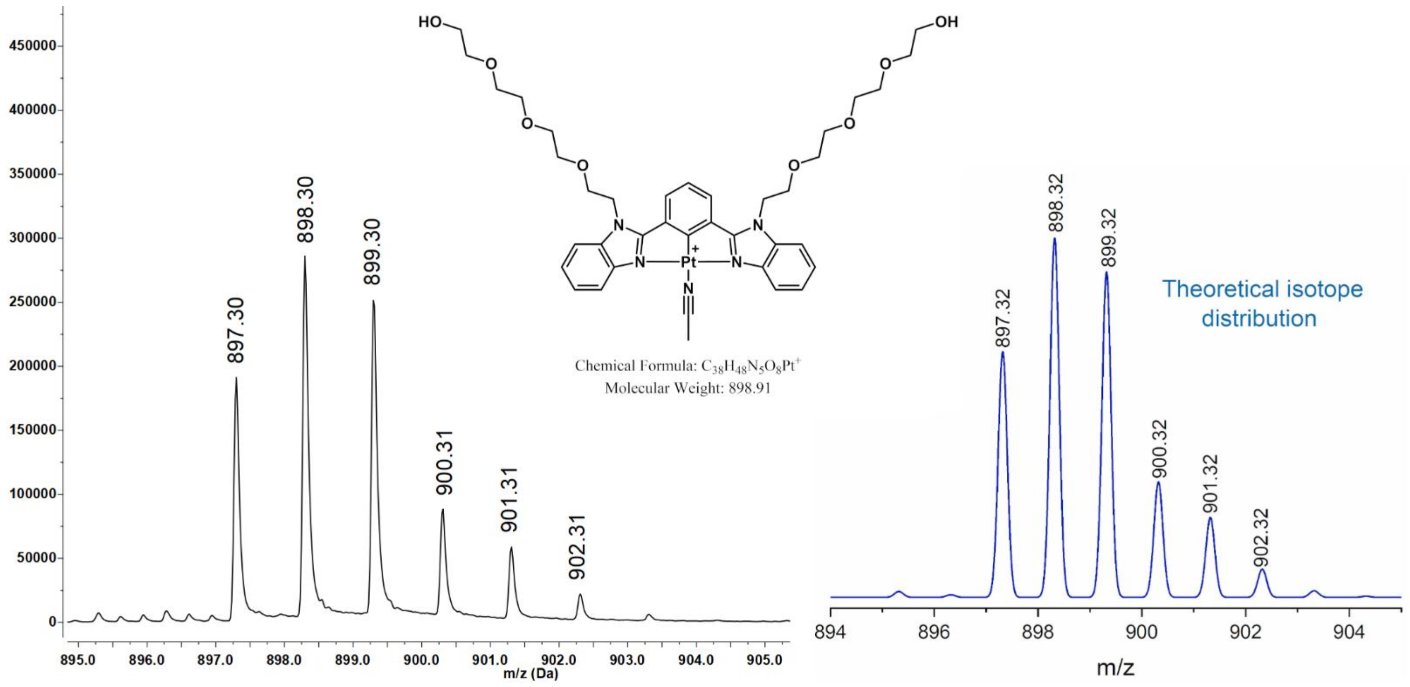

Figure S13. Positive scan ESI-MS spectrum of **4** in acetonitrile.

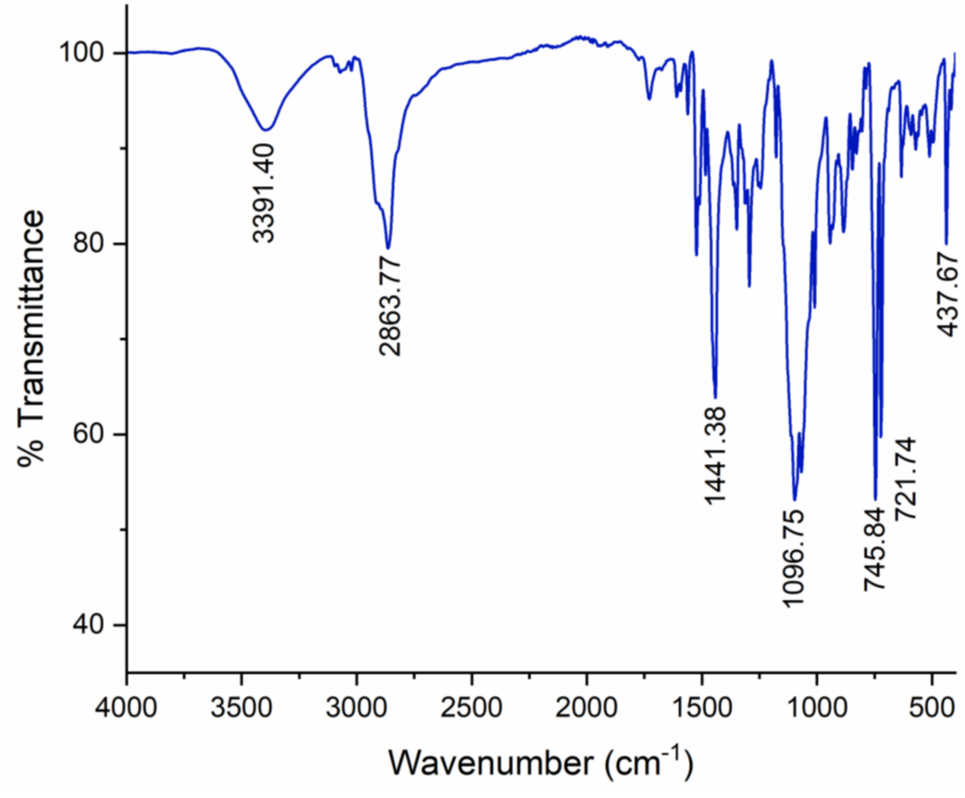

Figure S14. ATR-IR spectrum of **4**.

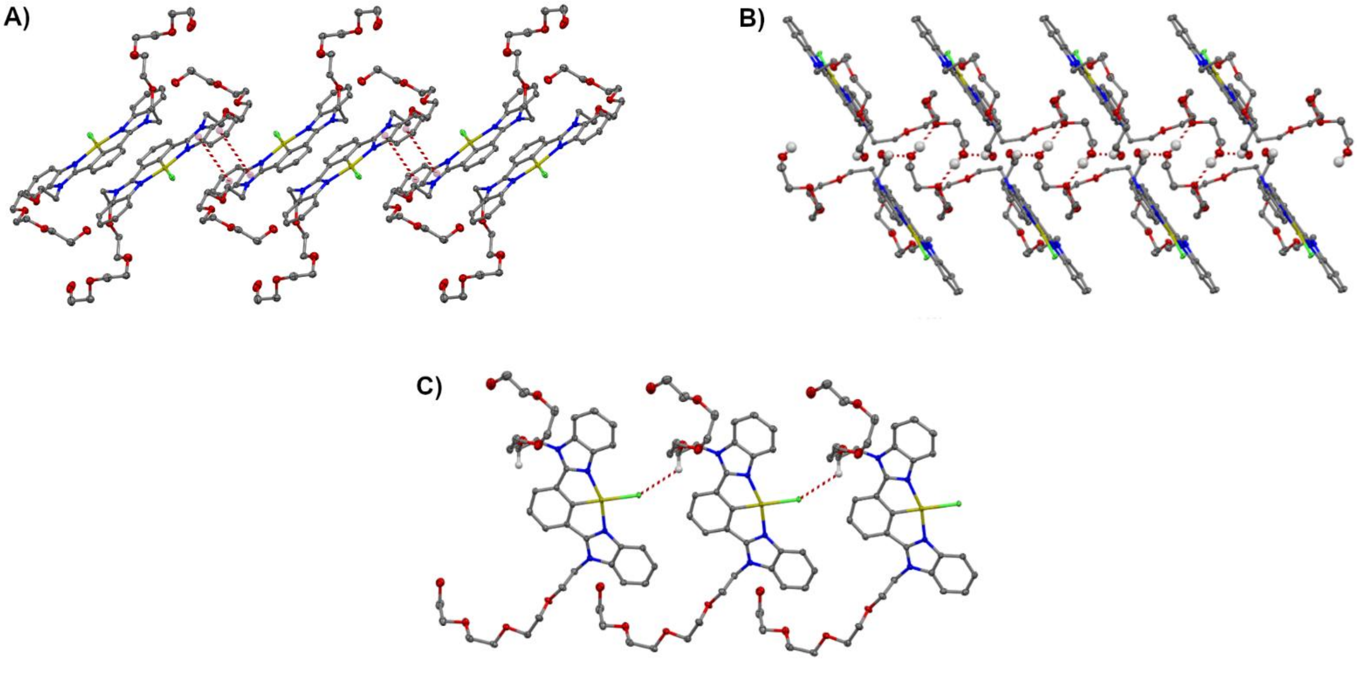

Figure S15. **A**). Dimers chain along the *b*-axis formed by π-π stacking interaction (3.67 Å). **B**) 1D chain along the *c*-axis induced by dimeric $R_2^2(10)$ homo-synthon. **C**) 1D chain via C-H···Cl interaction along the *a*-axis. Only the relevant hydrogen atoms are shown for clarity.

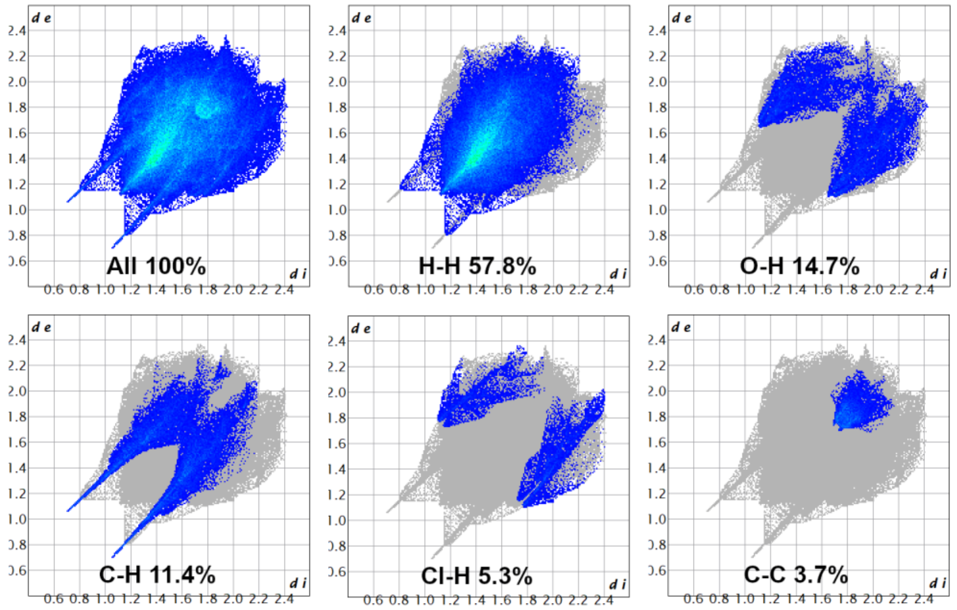

Figure S16. 2D-fingerprint plots of the complex **4** showing the contributions of reciprocal contacts to crystal packing.

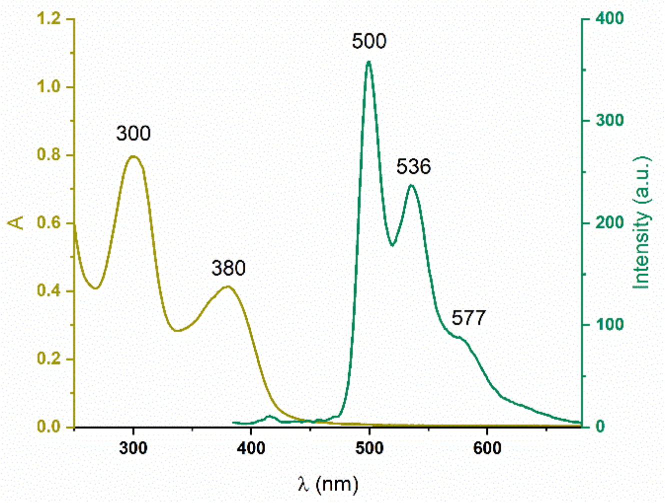

Figure S17. Absorption (15 μM) and emission (3 μM, λ_{exc}= 380 nm) spectra of **4** in aqueous MOPS/EtOH buffer solution (7:3, 10 mM, pH = 7.4).

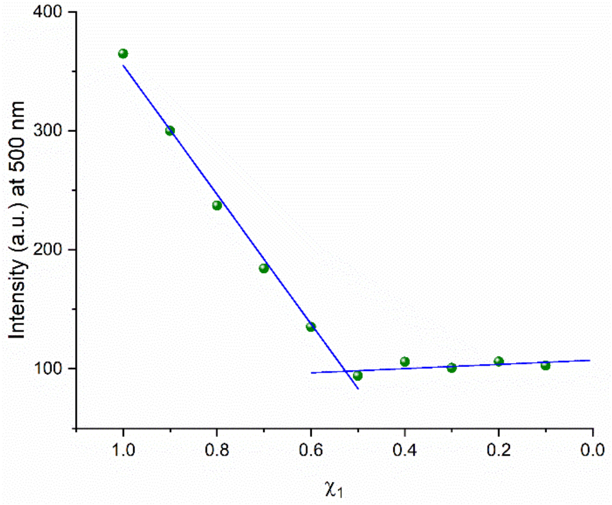

Figure S18. Job's plot between **4** (3 μM, λ_{exc}= 380 nm) and GTP in aqueous MOPS/EtOH buffer solution (7:3, 10 mM, pH = 7.4). Line-line intersection is $\chi \approx 0.5$ confirming the 1:1 binding model.

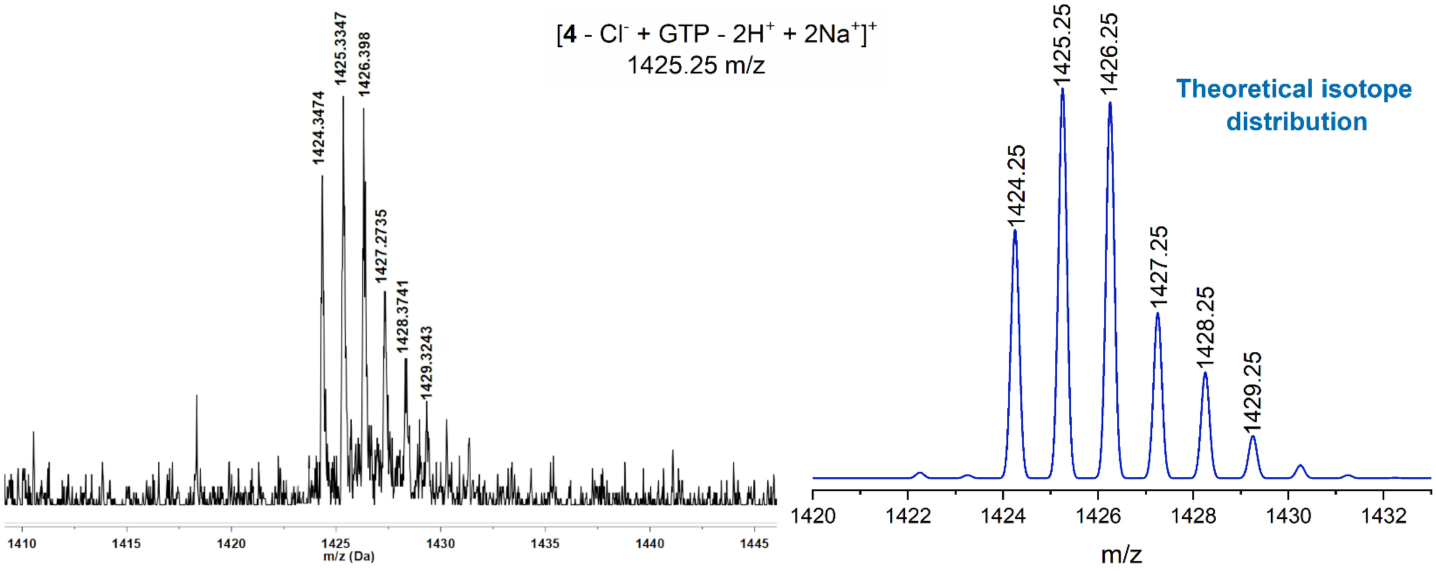

Figure S19. Species at 1425.3347 m/z found in the HRMS-ESI(+) spectrum of the resulting solution from UV-vis titration between receptor **4** and GTP, and the theoretical isotope distribution of the [**4** – Cl^- + GTP – $2H^+$ + $2Na^+$]$^+$.

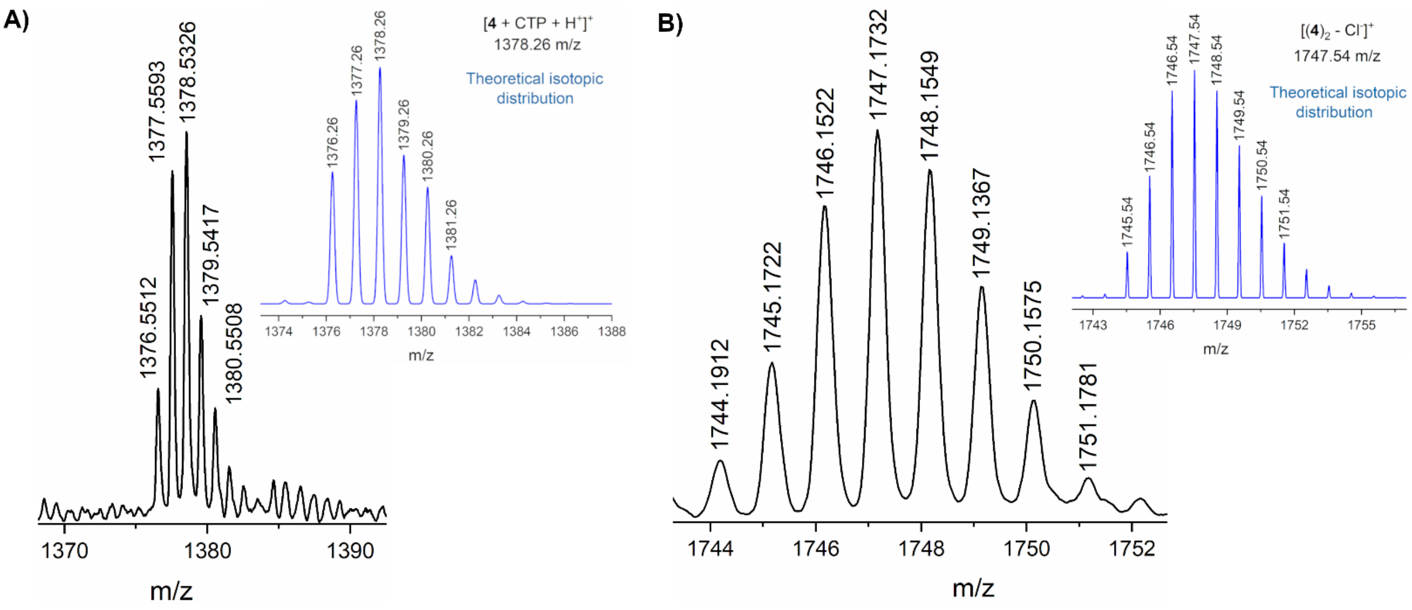

Figure S20. Species at **A**) 1378.5326 and **B**) 1747.1732 *m/z* found in the MALDI-TOF HRMS spectrum of the resulting solution from UV-vis titration between receptor **4** and CTP, and the theoretical isotopic distribution of the [**4** + CTP + H^+]$^+$ and [(**4**)$_2$ – Cl^-]$^+$.

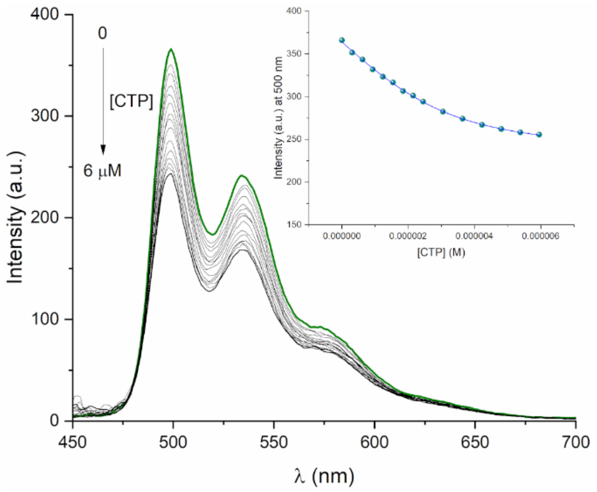

Figure S21. Changes in the emission spectra (λ_{exc} = 380 nm) of receptor **4** (3 μM) upon the addition of CTP (0 – 6 μM) in aqueous MOPS/EtOH buffer solution (7:3, 10 mM, pH = 7.4). The curve was fitted using equation (2).

Table S1. Crystallographic data for **4**.

	**4**
Empirical formula	$C_{36}H_{45}ClN_4O_8Pt$
Mol. Weight / g mol^{-1}	892.30
Crystal system	Triclinic
Space group	P-1
a / Å	9.7227(5)
b / Å	12.7654(6)
c / Å	16.1769(8)
α / °	111.614(2)
β / °	92.520(2)
γ / °	110.631(2)
Volume / Å^3	1711.71(15)
Z	2
Density / mg cm^{-3}	1.731
Temperature / *K*	150(2)
Abs. Coeff. / mm^{-1}	4.237
Θ range / °	1.869 to 27.503
Index ranges	-12<=h<=12, -16<=k<=16, -20<=l<=20
Reflections collected	97860
Max. & min. transmission	0.7455 and 0.4973
Data / restraints / param.	7827 / 21 / 467
Goodness-of-fit on F^2	1.170
Final *R* indices [I > 2 s (I)]	R1 = 0.0179, wR2 = 0.0432
R indices (all data)	R1 = 0.0193, wR2 = 0.0449
Larg. Diff. peak and hole / eÅ^{-3}	0.828 and -0.667

Table S2. Selected bond distances (Å) and angles (°) around Pt atoms in **4**.

	**4**
Pt(1)-C(10)	1.929(2)
Pt(1)-N(11)	2.0306(19)
Pt(1)-N(1)	2.0316(19)
Pt(1)-Cl(1)	2.4262(5)
C(10)-Pt(1)-N(11)	79.99(9)
C(10)-Pt(1)-N(1)	79.92(9)
N(11)-Pt(1)-N(1)	159.91(8)
C(10)-Pt(1)-Cl(1)	178.76(7)
N(11)-Pt(1)-Cl(1)	100.92(6)
N(1)-Pt(1)-Cl(1)	99.15(5)

Table S3. Hydrogen bonds for **4** [Å and °].

D-H…A	d(D-H)	d(H…A)	d(D…A)	<(DHA)
O(4)-H(4A)…O(3)#1	0.87(5)	1.92(5)	2.785(3)	167(4)
O(8)-H(8)…O(4)#2	0.78(4)	1.97(4)	2.740(3)	174(4)

Symmetry transformations used to generate equivalent atoms:

#1 -x+1,-y,-z+2 #2 x,y,z-1

Table S4. Absorption and emission maxima (nm) of **4** in aqueous MOPS/EtOH buffer solution (7:3, 10 mM, pH = 7.4).

Receptor	λ_{abs} (log ε)	λ_{em}[a]
**4**	300(4.72)<br>380(4.44)	500<br>536<br>577

[a]λ_{ex}= 380 nm

Mathematical Model to calculate the binding constant *$K_{1:1}$* an oligomerization process *K_M*

Binding constants were determined by a nonlinear least-squares of the absorbance vs the concentration of analyte according to the follow equation:

$$[H]+[G] \underset{}{\overset{K_{1:1}}{\rightleftharpoons}} [HG]+[HG] \overset{K_M}{\rightleftharpoons} [(HG)_2]+[HG] \overset{K_M}{\rightleftharpoons} [(HG)_3]+[HG] \overset{K_M}{\rightleftharpoons} \ldots$$

$$\frac{X-X_o}{X_{\lim}-X_o} = \frac{\delta\frac{[HG]}{1-K_M[HG]}}{\delta[H_O]} \qquad \text{equation S1}$$

X_o is the absorbance or emission intensity of MMLCT band in the absence of guest (X_o = 0). X_{lim} is the absorbance or emission intensity in presence of an excess of guest. X_{lim} is proportional to the concentration of H_o ($X_{lim} = \delta[H_o]$, δ is the proportionality constant of the complex in the aggregated state). Hence, the absorbance/intensity (X) can be expressed as:

$$\frac{X - X_o}{X_{\mathrm{lim}} - X_o} \approx \frac{2K_M(K_{1:1})^2[G]^2[H_o]}{1 + K_{1:1}[G] + 2K_M(K_{1:1})^2[H_o][G]^2} \qquad \text{equation S2}$$

$$A \approx A_o + (A_{lim} - A_o)\,\frac{2K_M(K_{1:1})^2[G]^2[H_o]}{1 + K_{1:1}[G] + 2K_M(K_{1:1})^2[H_o][G]^2} \qquad \text{equation S3}$$

Where A and A_o are the absorbance of the host in the absence and presence of the guest, respectively. $[H_o]$ is the concentration total of host. [G] is the concentration of guest. A_{lim} is the absorbance at saturation in the presence of excess guest. $K_{1:1}$ is the apparent binding constat for 1:1 model. K_M is the constant for the oligomerization of $[(HG)_\infty]$.

Cartesian coordinates of the geometry of complex **4-GTP**

15	2.588400	-2.556959	-3.534151
15	-0.324086	-1.774509	-2.983688
8	-0.906199	-2.330579	-4.285776
8	0.494254	-4.353776	0.030975
15	-0.181075	-3.033160	-0.287049
8	-0.248246	-3.080245	-1.948433
8	0.413332	-1.737645	0.195464
8	-1.757720	-3.035622	0.106408
6	-2.581832	-4.139290	-0.293592
6	-3.786441	-3.642953	-1.072796
8	-4.629649	-2.839722	-0.222732
6	-3.430568	-2.751968	-2.265826
8	-3.248566	-3.495469	-3.448038
6	-4.629470	-1.797981	-2.358675
8	-5.674231	-2.415532	-3.079676
6	-5.051948	-1.669898	-0.893718
7	-4.476344	-0.490153	-0.219655
6	-3.150543	-0.126743	-0.127902
7	-2.990256	0.848268	0.754947
6	-4.245665	1.139024	1.263004
6	-4.665126	2.104848	2.235818
8	-4.024553	2.939359	2.855619
7	-6.079445	1.998044	2.429410
6	-6.919073	1.116134	1.792348
7	-8.255514	1.209681	2.084864
7	-6.512308	0.241224	0.901870
6	-5.179598	0.300658	0.666168

1	-6.132725	-1.554612	-0.797055
1	-4.356555	-0.825042	-2.789672
1	-2.550767	-2.167407	-2.022122
1	-4.359203	-4.512111	-1.423169
1	-2.023971	-4.835388	-0.927120
1	-2.897476	-4.662877	0.611948
1	-6.456688	2.694273	3.060655
1	-2.347645	-0.560602	-0.715177
1	-8.519549	1.498770	3.015536
1	-8.799562	0.446586	1.707052
1	-5.207616	-2.995311	-3.708975
1	-2.443686	-3.130936	-3.892685
78	-1.086434	1.363793	1.648423
7	-0.849118	-0.364144	2.744668
6	0.694182	1.658691	2.377431
7	-0.635386	3.189518	0.822063
6	0.411906	-0.528302	3.192268
6	-1.523327	-1.555607	2.937135
6	1.308088	0.623300	3.110897
6	1.365228	2.866211	2.102832
6	0.586445	3.658961	1.158014
6	-1.140910	3.993280	-0.178021
7	0.599334	-1.802524	3.653619
6	-2.852888	-1.914300	2.706145
6	-0.607443	-2.486202	3.474750
6	2.582250	0.837482	3.653367
6	2.614526	3.094642	2.701478
7	0.892286	4.760084	0.401944
6	-2.339589	3.915427	-0.891435
6	-0.184148	4.992341	-0.459804
6	1.899701	-2.470853	3.761865
6	-3.223239	-3.222248	2.988946
1	-3.563316	-1.200869	2.314826
6	-0.971016	-3.805671	3.738973
1	3.124762	0.073475	4.196385
6	3.197990	2.083223	3.469108
1	3.132470	4.041197	2.594973
6	2.164436	5.469981	0.321469
6	-2.553264	4.870517	-1.877553
1	-3.053912	3.131026	-0.673976
6	-0.400589	5.952173	-1.447238
1	1.750007	-3.425442	4.268861
1	2.581707	-1.879341	4.371659
6	2.412172	-2.662891	2.336918
6	-2.294188	-4.157497	3.484378
1	-4.243200	-3.533893	2.791748
1	-0.261135	-4.529811	4.122356
1	4.166677	2.258129	3.925114
6	3.321320	4.628364	-0.219107
6	-1.601552	5.874411	-2.146306
1	-3.470664	4.843889	-2.456527
8	3.721380	-3.238456	2.336387
1	1.724571	-3.308921	1.785257
1	2.432434	-1.692195	1.839280

1	-2.615151	-5.176393	3.675878
8	2.929984	3.979956	-1.406269
1	4.177536	5.303582	-0.381625
1	3.626015	3.878353	0.519966
1	-1.803948	6.601149	-2.926226
6	4.142485	-3.534709	0.993654
6	3.897550	3.038296	-1.880009
1	3.261299	-3.724246	0.373724
1	4.741215	-4.451287	1.035328
6	4.990093	-2.436460	0.381750
1	4.479817	2.664761	-1.027764
1	4.588527	3.531437	-2.579099
6	3.210338	1.873883	-2.573340
8	4.255342	-1.202544	0.404619
1	5.916011	-2.306863	0.946845
1	5.210961	-2.707432	-0.658434
1	2.659350	2.234464	-3.441766
1	3.972338	1.172115	-2.928725
8	2.365875	1.140642	-1.692951
6	5.061816	-0.040079	0.231343
6	0.996382	1.585188	-1.555597
1	4.384182	0.735190	-0.136546
1	5.833549	-0.216889	-0.530463
6	5.676318	0.444694	1.529645
6	0.287315	1.897584	-2.858209
8	6.754296	-0.399308	1.918026
1	6.038448	1.479173	1.388812
1	4.897603	0.452978	2.294305
8	0.554224	3.259099	-3.191667
6	7.051111	-0.355011	3.307840
6	0.511753	3.580864	-4.564379
1	6.765013	0.618419	3.731676
1	8.138170	-0.459899	3.415272
6	6.357605	-1.477888	4.073449
1	-0.509201	3.504798	-4.972803
1	1.159548	2.894915	-5.136084
6	1.031701	5.010715	-4.668367
8	4.945634	-1.395206	3.990148
1	6.713022	-2.443717	3.688391
1	6.652939	-1.416885	5.130392
8	2.268253	5.163394	-3.999082
1	1.187859	5.274575	-5.720098
1	0.269351	5.691358	-4.260067
1	4.639794	-2.019281	3.300950
1	2.161763	4.766166	-3.117327
1	0.336591	6.708274	-1.692470
1	2.435146	5.861156	1.307272
1	1.998698	6.327202	-0.331386
1	0.632220	1.214877	-3.644931
1	-0.786972	1.734730	-2.731093
1	0.981820	2.472384	-0.930491
1	0.471604	0.771980	-1.052913
8	-1.039140	-0.644653	-2.298378
8	1.231766	-1.429529	-3.181976

8	1.866229	-3.823441	-4.054217
8	3.130904	-2.785774	-2.113184
8	3.326589	-1.761065	-4.576203
11	2.283204	-1.033794	-0.933382
11	0.871443	-2.633467	-5.651361
11	1.601775	-4.632492	-1.960228

Cartesian coordinates of the geometry of complex **4-CTP**

15	4.774666	-1.166533	-2.672104
15	1.981356	-2.467396	-2.393408
8	2.215621	-3.814845	-3.085014
8	3.326010	-2.213978	1.455182
15	2.097429	-1.993706	0.591169
8	2.559215	-2.691581	-0.841739
8	1.557021	-0.603568	0.388506
8	0.868493	-2.952376	1.045624
6	1.105210	-4.358835	1.142118
6	-0.025986	-5.110881	0.479669
8	-1.248736	-4.891175	1.211558
6	-0.318268	-4.693230	-0.968550
8	0.433148	-5.476794	-1.870389
6	-1.827441	-5.004021	-1.114592
8	-1.998399	-6.321252	-1.596979
6	-2.345947	-4.969724	0.335992
6	-4.833557	-3.091836	2.296089
6	-4.132077	-1.857393	2.475203
7	-4.380881	-0.977029	3.448762
7	-3.215217	-1.549157	1.538609
6	-2.767059	-2.442680	0.566160
8	-1.979338	-2.169176	-0.303452
7	-3.261330	-3.844713	0.671844
6	-4.177240	-3.905465	1.610908
1	-2.342075	-4.271968	-1.746704
1	-0.152276	-3.625546	-1.105751
1	0.212196	-6.183999	0.488781
1	2.031143	-4.639243	0.630759
1	1.188309	-4.627410	2.198435
1	-1.171798	-6.489731	-2.089772
1	1.037806	-4.896525	-2.399136
78	-2.204105	0.398952	1.556665
7	-0.605065	-0.050992	2.760518
6	-1.293609	2.124595	1.579305
7	-3.461222	1.505573	0.360441
6	0.297946	0.950024	2.847970
6	-0.076250	-1.154570	3.400556
6	-0.074916	2.239332	2.271113
6	-1.878567	3.215466	0.911890

6	-3.050381	2.778604	0.160528
6	-4.558101	1.278384	-0.444545
7	1.407154	0.535203	3.520337
6	-0.594321	-2.433027	3.621273
6	1.209067	-0.803036	3.867948
6	0.539638	3.496355	2.353953
6	-1.302417	4.484587	1.076704
7	-3.837236	3.387315	-0.778365
6	-5.359703	0.145542	-0.605964
6	-4.815280	2.462952	-1.165444
6	2.702083	1.218867	3.542037
6	0.200786	-3.332255	4.318134
1	-1.557742	-2.721617	3.226365
6	2.020133	-1.715678	4.540670
1	1.496144	3.656793	2.837055
6	-0.104600	4.603230	1.785064
1	-1.781313	5.376953	0.692066
6	-3.619060	4.639474	-1.505966
6	-6.430738	0.242635	-1.486385
1	-5.144838	-0.764485	-0.060740
6	-5.894436	2.563648	-2.041986
1	3.334156	0.714922	4.274509
1	2.575587	2.250192	3.873673
6	3.314378	1.132344	2.145565
6	1.491073	-2.981772	4.764641
1	-0.169259	-4.336480	4.496604
1	3.023067	-1.461286	4.861816
1	0.348287	5.582645	1.898815
6	-2.186251	4.856483	-2.012669
6	-6.696493	1.434421	-2.188469
1	-7.076435	-0.616215	-1.637311
8	4.561633	1.831381	2.131620
1	3.465690	0.081463	1.888586
1	2.627593	1.561468	1.413064
1	2.092352	-3.720871	5.283699
8	-1.634239	3.622875	-2.405155
1	-2.241793	5.555964	-2.861447
1	-1.549743	5.318237	-1.251601
1	-7.543111	1.474352	-2.865794
6	5.328975	1.528867	0.953191
6	-0.274047	3.743492	-2.809979
1	5.102441	0.519299	0.605844
1	6.385319	1.579553	1.239549
6	5.101017	2.503982	-0.184469
1	0.313752	4.146340	-1.972272
1	-0.199364	4.446674	-3.655451
6	0.285601	2.398136	-3.245434
8	3.711186	2.511135	-0.555732
1	5.390611	3.515064	0.111493
1	5.703401	2.173393	-1.039936
1	-0.383896	1.951100	-3.985856
1	1.246970	2.586029	-3.737570
8	0.555696	1.503300	-2.181379
6	3.300407	3.696648	-1.226498

6	-0.567326	0.737245	-1.663818
1	2.402940	3.425907	-1.787532
1	4.067342	4.027684	-1.939990
6	2.940471	4.819537	-0.270333
6	-1.369040	0.008353	-2.722494
8	4.122400	5.425766	0.235332
1	2.331490	5.568753	-0.807812
1	2.334395	4.403609	0.539397
8	-2.440169	0.857022	-3.162901
6	3.934324	6.198731	1.413591
6	-2.906022	0.599163	-4.470951
1	2.882334	6.501852	1.513037
1	4.537485	7.110838	1.314542
6	4.371536	5.434960	2.658475
1	-3.324714	-0.415089	-4.566114
1	-2.075484	0.689517	-5.191889
6	-3.977386	1.646222	-4.746369
8	3.596038	4.271890	2.878668
1	5.436770	5.182301	2.564000
1	4.266049	6.094367	3.531111
8	-3.513642	2.950850	-4.431784
1	-4.255641	1.626315	-5.806711
1	-4.869226	1.396986	-4.156403
1	4.056455	3.497064	2.492043
1	-2.889445	2.845544	-3.690671
1	-6.098280	3.466378	-2.604880
1	-3.925751	5.497346	-0.897758
1	-4.273714	4.581860	-2.374752
1	-0.720956	-0.280367	-3.555912
1	-1.772631	-0.911044	-2.292119
1	-1.224512	1.410469	-1.115013
1	-0.116791	0.018545	-0.982467
8	0.606703	-1.890044	-2.350586
8	3.026821	-1.385970	-2.981838
8	5.247298	-2.624620	-2.423937
8	4.721777	-0.379685	-1.348313
8	5.214149	-0.525474	-3.953615
11	2.564349	0.527052	-1.366358
11	4.143742	-3.433383	-4.169712
11	4.863274	-2.431814	-0.204480
1	-5.103455	-1.164040	4.124659
1	-3.858428	-0.104647	3.457531
1	-2.943621	-5.869428	0.515838

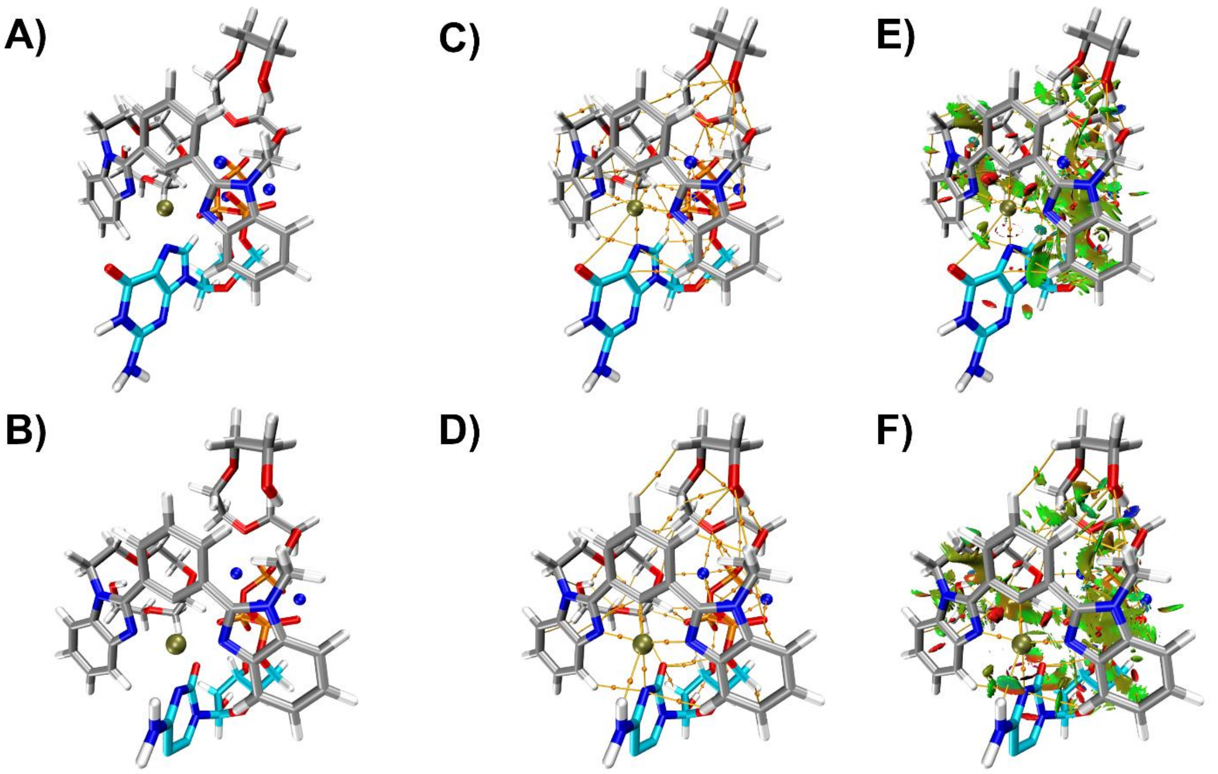

Figure S21. A, B) Molecular graphs obtained from geometry optimization at B3LYP DFT-D3(BJ) 6-31G(d,p)/LANL2DZ/MWB60 level of theory; C, D) AIM molecular geometries with bond critical points (BCPs) and bond paths (BPs) of non-covalent interactions, and; E, F) NCI isosurfaces together with AIM CPs and BPs. Upper row corresponds to **4-GTP** and lower row to **4-CTP**. AIM and NCI analysis were performed using Multiwfn.

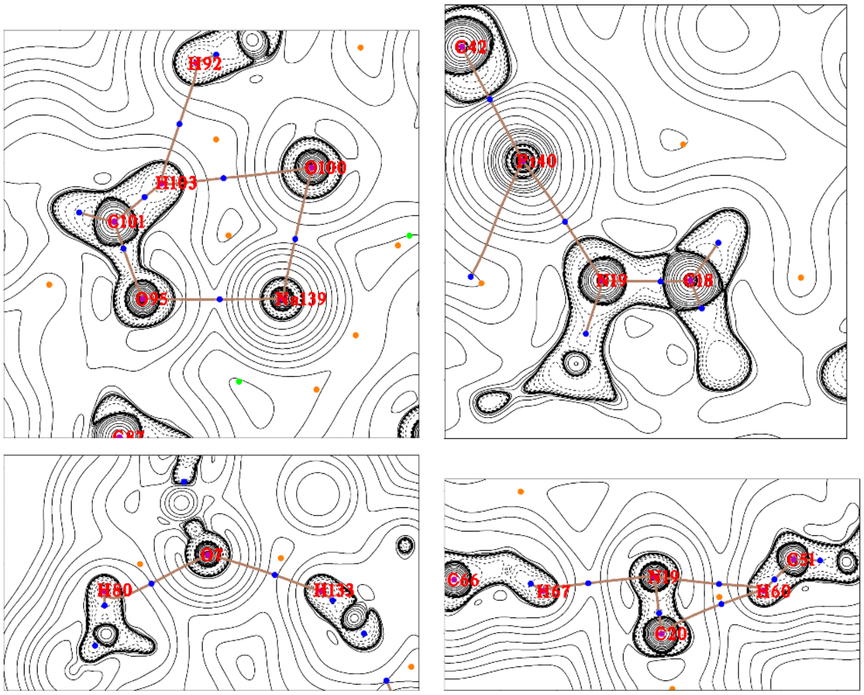

Figure S22. Plots of contour lines of the Laplacian of electron density for the 1:1 GTP–Pt-NCN complex, **4-GTP**, obtained at B3LYP DFT-D3(BJ) 6-31G(d,p)/LANL2DZ/MWB60 level of theory. Brown lines represent bond paths (BPs); blue and orange dots represent bond critical points (BCPs) and ring critical points, respectively. Plots were obtained using Multiwfn's built-in QTAIM plotting routine. Atom labels correspond to cartesian coordinates in input file.

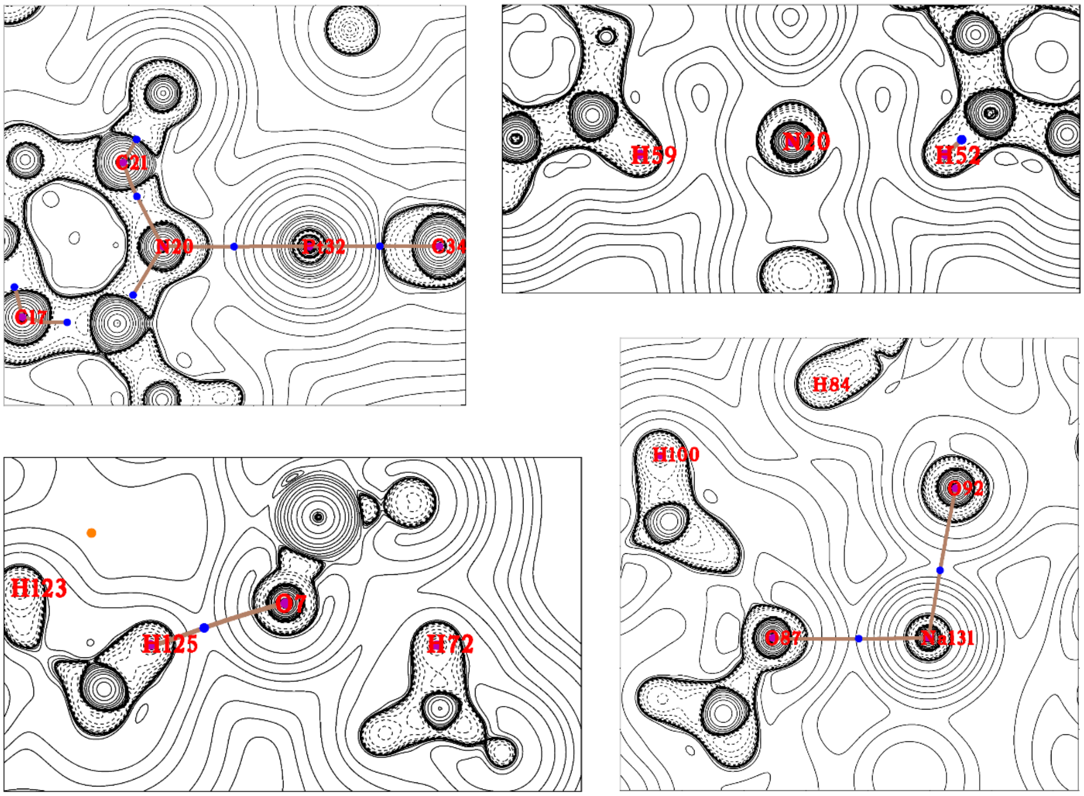

Figure S23. Plots of contour lines of the Laplacian of electron density for the 1:1 CTP–Pt-NCN complex, **4-CTP**, obtained at B3LYP DFT-D3(BJ) 6-31G(d,p)/LANL2DZ/MWB60 level of theory. Brown lines represent bond paths (BPs); blue and orange dots represent bond critical points (BCPs) and ring critical points, respectively. Plots were obtained using Multiwfn's built-in QTAIM plotting routine. Atom labels correspond to cartesian coordinates in input file.
